# Preneoplastic liver colonization by 11p15.5 altered mosaic cells in young children with hepatoblastoma

**DOI:** 10.1038/s41467-023-42418-9

**Published:** 2023-11-06

**Authors:** Jill Pilet, Theo Z. Hirsch, Barkha Gupta, Amélie Roehrig, Guillaume Morcrette, Aurore Pire, Eric Letouzé, Brice Fresneau, Sophie Taque, Laurence Brugières, Sophie Branchereau, Christophe Chardot, Isabelle Aerts, Sabine Sarnacki, Monique Fabre, Catherine Guettier, Sandra Rebouissou, Jessica Zucman-Rossi

**Affiliations:** 1 Centre de Recherche des Cordeliers, Université Paris Cité, Sorbonne Université, Inserm, F-75006 Paris, France; 2 Gustave Roussy, Université Paris-Saclay, Department of Children and Adolescents Oncology, Villejuif, France; 3 Department of Paediatrics, CHU Rennes, Rennes, France; 4 Department of Pediatric Surgery, Bicêtre Hospital, AP-HP, Paris-Saclay University, Le Kremlin-Bicêtre, France; 5 Department of Pediatric Surgery, Hôpital Necker-Enfants Malades, AP-HP, Université Paris Cité, Paris, France; 6 Institut Curie, PSL Research University, Oncology Center SIREDO, Paris, France; 7 Pathology Department, Necker Enfants Malades Hospital, Université Paris Cité, AP-HP, Paris, France; 8 Department of Pathology Hôpital Bicêtre–AP-HP, INSERM U1193, Paris-Saclay University, Le Kremlin-Bicêtre, France; 9 Institut du Cancer Paris CARPEM, AP-HP, Department of Oncology, Hopital Européen Georges Pompidou, F-75015 Paris, France

**Keywords:** Paediatric cancer, Epigenomics, Embryonal neoplasms, Liver cancer

## Abstract

Pediatric liver tumors are very rare tumors with the most common diagnosis being hepatoblastoma. While hepatoblastomas are predominantly sporadic, around 15% of cases develop as part of predisposition syndromes such as Beckwith-Wiedemann (11p15.5 locus altered). Here, we identify mosaic genetic alterations of 11p15.5 locus in the liver of hepatoblastoma patients without a clinical diagnosis of Beckwith-Wiedemann syndrome. We do not retrieve these alterations in children with other types of pediatric liver tumors. We show that mosaic 11p15.5 alterations in liver FFPE sections of hepatoblastoma patients display *IGF2* overexpression and *H19* downregulation together with an alteration of the liver zonation. Moreover, mosaic livers’ microenvironment is enriched in extracellular matrix and angiogenesis. Spatial transcriptomics and single-nucleus RNAseq analyses identify a 60-gene signature in 11p15.5 altered hepatocytes. These data provide insights for 11p15.5 mosaicism detection and its functional consequences during the early steps of carcinogenesis.

## Abstract (web summary)

Paediatric liver cancer is rare, and often associated with a predisposition syndrome. Here, the authors show that 11p15.5 mosaic alteration in the liver is a pre-neoplastic lesion associated with hepatoblastoma, and spatial transcriptomics together with single-nucleus RNAseq identify a an altered zonation in the liver of these patients.

## Introduction

Pediatric liver cancers (PLCs) are rare and account for only 1–2% of all pediatric malignancies. Of these, the most frequent tumor is hepatoblastoma (HB) which affects mainly young infants before 5 years old. While HB is mostly sporadic, 15% of HB cases are associated with a predisposition syndrome such as familial adenomatous polyposis (FAP), trisomy 18, Simpson-Golabi-Behmel syndrome or Beckwith-Wiedemann syndrome (BWS), an overgrowth syndrome caused by a genetic or epigenetic alteration at locus 11p15.5^1^. Cardinal features of BWS are notably macroglossia, exomphalos, lateralized overgrowth, and hyperinsulinism^2^ but BWS is a heterogeneous disease with a spectrum of clinical features. Indeed, most BWS patients harboring 11p15.5 molecular defect do not display all phenotypic features of BWS. Pediatric hepatocellular carcinoma (pHCC) is another type of pediatric liver cancer, accounting for approximately 20% of diagnosed PLC. pHCC occurs predominantly in adolescents and young adults. They develop either sporadically or in the context of an underlying chronic liver disease due to hepatitis B virus (HBV) or associated with rare inherited metabolic syndromes^3,4^. Pediatric HCC often presents a deletor phenotype with *AMER1*, *GPC3*, *RPS6KA3* deletions as well as MAPK pathway activation^5–7^. Fibrolamellar carcinoma is a rare subtype of HCC occurring in the absence of underlying etiology, and characterized by large oncocytic neoplastic cells separated by collagen bundles in histology and a recurrent *DNAJB1*-*PRKACA* fusion transcript^8^. Finally, pediatric benign hepatocellular tumors are rare, mainly represented by hepatocellular adenomas (pHCA) and correspond to less than 5% of PLCs. The average onset of HCAs in children is around 14 years old, they can be associated with metabolic diseases or congenital malformations related to vascular anomalies^9^.

Genomic analyses of large series of HB showed that almost all of them (~70–90%) carry a *CTNNB1* alteration encoding for β-catenin^6,10–12^. The second most frequently altered locus is 11p15.5 locus in up to 84 % of HB and 89% of pHCC patients^6,13,14^. 11p15.5 locus is a parentally imprinted locus and contains key growth regulator genes such as *IGF2* oncogene, *H19* non-coding RNA, and *CDKN1C* tumor suppressor as illustrated in Supplementary Fig. 1. A precise dosage balance of these genes is crucial for maintaining normal liver development and tissue homeostasis and is controlled through two Imprinting Center regions, IC1 and IC2 that contain tandem repeats submitted to methylation. *IGF2* and *H19* share the same enhancers and their expression is regulated through IC1 methylation status. IC1 is methylated on the paternal allele and unmethylated on the maternal allele resulting in *IGF2* expression from the paternal inherited allele and *H19* from the maternal allele^15–18^. On the other hand, IC2 is unmethylated on the paternal allele leading to the transcription of *KCNQ1OT1*, a non-coding regulatory RNA able to repress *CDKN1C* and *KCNQ1* expression. Conversely, the maternal allele is methylated on IC2 and expresses the tumor suppressor *CDKN1C* and *KCNQ1*^19,20^. Also, *IGF2* is expressed by only one allele through P2-P3-P4 promoters during fetal development and progressively switches to a biallelic expression through P1, a liver-specific promoter after birth. Since IC1 and IC2 control the expression of very important genes for growth during development (especially *IGF2*, *H19,* and *CDKN1C*), a change in IC1, IC2 methylation levels or chromosome alterations such as copy-neutral loss of heterozygosity (cn-LOH) at locus 11p15.5 can induce meaningful gene expression changes. Early post-zygotic modifications during development can result in high mosaicism in individuals. We recently identified a mosaic predisposing 11p15.5 locus alteration in 6 non-tumor liver tissues from HB patients, occurring prior to β-catenin transforming mutation.

To characterize pre-malignant expansions with 11p15.5 locus defects in normal liver, here we analyze a cohort of 131 patients with PLC (Supplementary Fig. 2). Detection of 11p15.5 locus mosaicism is carried out using whole-exome and whole-genome sequencing (WES, WGS), multiplex ligation-dependent probe amplification (MS-MLPA) methylation analysis, and RNAscope in situ hybridization. We investigate the functional consequences of the mosaic 11p15.5 alterations using RNAseq, spatial transcriptomics, and single-nucleus RNAseq (snRNAseq) analyses to better understand their role in early liver carcinogenesis.

## Results

### Molecular characterization of pre-malignant mosaic 11p15.5 expansions

**Fig. 1:**
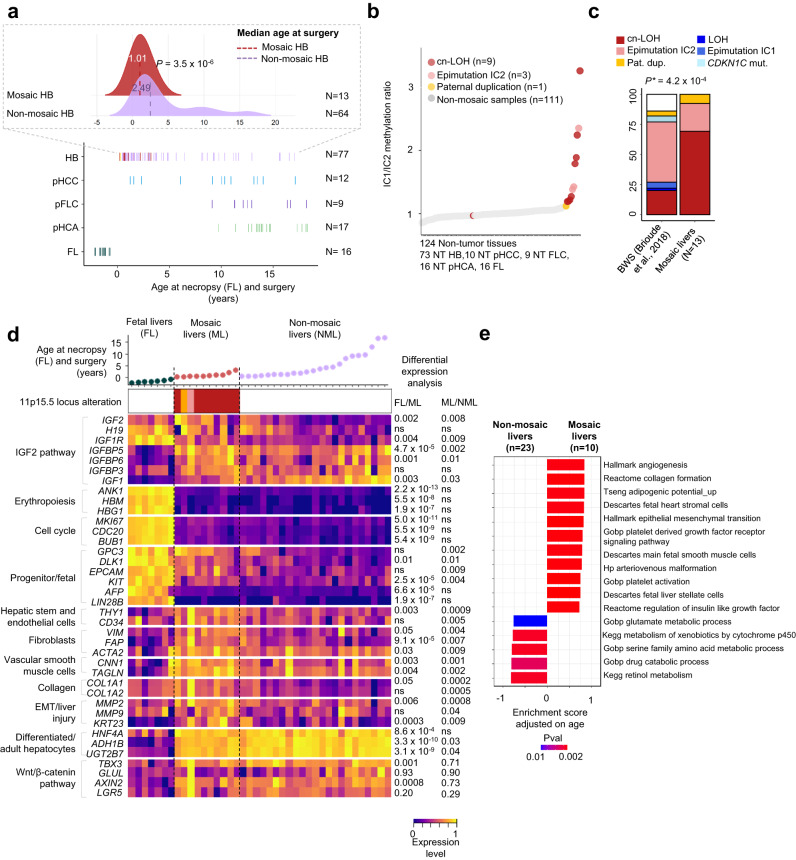
Molecular features of 11p15.5 locus pre-malignant expansions. **a** Bottom: age at necropsy (fetal livers) and surgery in 131 patients with pediatric liver tumors. Patients with mosaicism are colored by type of 11p15.5 alteration (cn-LOH: red, paternal duplication: orange, epimutation IC2: light red). Top: median age comparison between 13 mosaic HB (red) and 64 non-mosaic HB (pink). Two-sided Student’s *t*-test was performed. **b** IC1/IC2 methylation ratio in 124 non-tumor tissues from patients with pediatric liver tumors. **c** Spectra of 11p15.5 locus alteration in 13 mosaic livers and patients with Beckwith-Wiedemann syndrome^2^. Two-sided *χ*² goodness of fit test was performed. **d** Heatmap representation of key gene expression in 7 fetal livers, 10 mosaic, and 23 non-mosaic livers. All *IGF2* isoforms were taken into account including P0 to P4 promoters. Limma differential expression analysis was performed adjusted for age at surgery. **e** Gene-set enrichment analysis using *fgsea* R package based on the adaptive multilevel splitting Monte Carlo approach was used to assess the significance of gene-set enrichment in 10 mosaic vs. 23 non-mosaic livers. ns: non-significant. NT: non-tumor, HB: hepatoblastoma, pHCC: pediatric hepatocellular carcinoma, pFLC: pediatric fibrolamellar carcinoma, pHCA: pediatric hepatocellular adenoma, FL: fetal liver, ML: Mosaic liver, NML: non-mosaic liver, LOH: loss of heterozygosity, cn-LOH: copy-neutral LOH, pat.dup: 11p15.5 paternal duplication. Source data are provided as a Source Data file.

To identify patients with pre-malignant expansions with 11p15.5 alteration, we screened non-tumor tissues from 115 patients (110 livers, 9 blood, 1 lung samples) with PLC for chromosome 11p15.5 copy-number alterations and IC1/IC2 methylation status using WES, WGS, and MS-MLPA (Fig. 1, Supplementary Fig. 3–5, Supplementary Data 1). We identified alterations at 11p15.5 locus exclusively in the liver of patients with HB (13/77, ~17%) but no liver mosaic 11p15.5 alteration was found in 16 Fetal livers (FL) and in 38 patients with pediatric HCC (0/12), FLC (0/9) or HCA (0/17). In addition, we analyzed the methylation levels of IC1 and IC2 in 14 fetal livers from the literature^21^. All 14 fetal liver samples displayed an average methylation of ~0.6 for both IC1 and IC2 corresponding to an average IC1/IC2 methylation ratio of 1 and indicating an absence of 11p15.5 mosaicism in these 14 fetal livers (Supplementary Fig. 6). Among the 13 mosaic patients, 9 carried a cn-LOH leading to *IGF2* expression by two paternal chromosomes, 3 had a loss of methylation at imprinting center 2 (epimutation IC2) and 1 patient showed a paternal duplication at 11p15.5 locus. In mosaic liver tissues, the fraction of cells carrying the 11p15.5 alteration ranged from 3% to 58%. All patients with 11p15.5 locus mosaicism were below 3.3 years old at diagnosis and globally younger than HB patients without 11p15.5 mosaic alteration (Fig. 1a, *P* = 3.5 x 10^−6^, Student’s *t*-test). In addition, they showed a loss of parental imprinting at 11p15.5 with IC1 hypermethylation and/or IC2 hypomethylation. Interestingly, IC1/IC2 methylation ratio was able to discriminate mosaic from normal liver samples in the overall cohort of 124 patients, except for patient #3131 for which only lung frozen tissue was analyzable that showed 3% of mosaic cells with cn-LOH (Fig. 1b).

Only one patient (#3180), was clinically diagnosed with Beckwith-Wiedemann syndrome and demonstrated the same IC2 epimutation in the liver and blood cells. In 4 other HB patients with 11p15.5 mosaicism in the liver and available blood samples, no 11p15.5 alteration was identified in the blood cells, in agreement with the lack of clinical BWS (Supplementary Figs. 3 and 4). We identified different alteration spectra of 11p15.5 alterations between our 13 mosaic livers from HB patients and overall BWS patients^2^. Notably, IC2 epimutation was the most frequent alteration in BWS while cn-LOH was predominant in mosaic livers (*P* = 4.2 x 10^−4^, Fig. 1c).

### Mosaic 11p15.5 livers display high *IGF2* gene expression, extracellular matrix, and angiogenesis markers expression

To better understand the transcriptomic deregulations related to 11p15.5 alterations in mosaic livers, we compared the overall transcriptomic profiles of different pediatric liver tissues (7 fetal, 10 mosaic, 23 non-mosaic livers) considering patient age at sampling (Fig. 1d; Supplementary Data 2 and 3). In mosaic livers, we identified an overexpression of genes belonging to IGF pathway (*IGF2*, *IGF1R*, *IGFBP5*, *IGFBP6*). *IGF2*, coding for insulin growth factor type 2, is an oncogenic growth factor and its overexpression is a hallmark of 11p15.5 altered hepatocytes^6,14^. *IGF2* was overexpressed in mosaic livers in relation to the transcription of two copies with similar usage of the *IGF2* P2-P3-P4 fetal promoters (corresponding to the paternal allele) compared with non-mosaic livers (Fig. 1d and Supplementary Fig. 7). In non-mosaic livers, we observed a switch from fetal to adult P0-P1 promoters’ usage due to progressive fetal promoter methylation during the first year of age. Interestingly, in mosaic livers, *IGF2* overexpression is associated with the persistence of *IGF1R* overexpression after birth and an abnormal overexpression of *IGFBP5* and *IGFBP6* leading to a coordinated overexpression of the insulin growth signaling pathway. In contrast, *IGF1* is downregulated in mosaic livers (*P* = 0.03).

Mosaic livers also demonstrated an overexpression of fibroblasts (*VIM*, *ACTA2*, *FAP*) and progenitor/stem cell (*GPC3*, *DLK1*, *EPCAM*, *THY1*, *KIT*) markers. Several of these genes are also expressed in fetal livers, however, in mosaic, genes involved in hematopoiesis (*ANK1*, *HBM*, *HBG1*), cell cycle (*MKI67*, *CDC20*, *BUB1*), and other fetal liver markers (*LIN28B*, *AFP*) were not overexpressed. Of note, mosaic livers did not display overexpression of the Wnt/β-catenin pathway targets (*TBX3*, *GLUL*, *AXIN2*, *LGR5*, Fig. 1d, Supplementary Fig. 8a). Moreover, *IGF2* expression levels did not correlate with Wnt/β-catenin target genes expression suggesting a lack of Wnt pathway activation in mosaic livers (Supplementary Fig. 8b). Gene-set enrichment analyses (GSEA) comparing non-mosaic and mosaic livers revealed enrichment in angiogenesis, stellate cells, epithelial-to-mesenchymal transition (EMT), platelet activation, vascular smooth muscle cells, and extracellular matrix signaling pathways in mosaic livers. Conversely, pathways of xenobiotics, glutamate, retinol, and amino acid metabolism were decreased (Fig. 1e and Supplementary Data 4). Altogether these results suggest that 11p15.5 mosaic liver tissues are enriched in extracellular matrix, EMT, angiogenesis, and retain a high *IGF2* pathway transcriptomic profile.

### Spatial and longitudinal heterogeneity in 11p15.5 mosaic livers

**Fig. 2:**
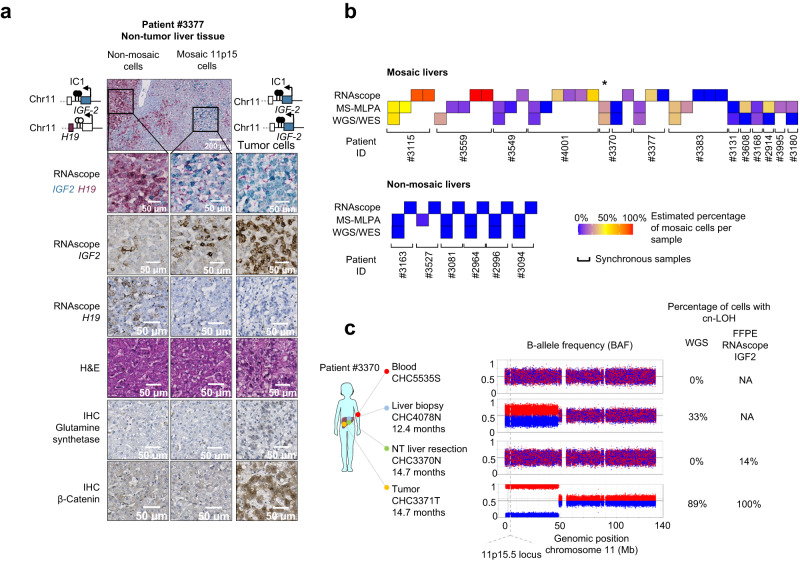
Spatial and longitudinal heterogeneity of 11p15.5 locus mosaic hepatocytes. **a** Representative non-mosaic, mosaic 11p15.5 cn-LOH, and tumor areas of FFPE slides from mosaic patient #3377. Six stainings were performed: singleplex and duplex RNAscope *IGF2*/*H19*, Hematoxylin and Eosin, β-catenin, and glutamine synthetase (GS) immunostainings. All *IGF2* isoforms (P0 to P4 promoters) were considered in *IGF2* singleplex and duplex RNAscope assay. In duplex RNAscope assay, 11p15.5 mosaic cells display *IGF2* overexpression (in blue) and *H19* expression loss (in red). Two slides from patient #3377 were analyzed. Scale bars, 50 µM and 200 µM. **b** Fraction of mosaic cells (%) in multiple samplings from the same patient using RNAscope *IGF2*/*H19*, MS-MLPA and WGS/WES detection methods. All samplings are synchronous samples collected at surgery except one liver biopsy labeled with an asterisk (*). **c** WGS-derived B-allele frequency (BAF) of heterozygous single-nucleotide polymorphisms (SNP) at chromosome 11 in mosaic patient #3370. We identified 33% of mosaic 11p15.5 cells in the liver biopsy but not in the blood or liver resection. *IGF2*/*H19* RNAscope assay allowed for the detection of 14% of mosaic cells in the FFPE section from liver resection. Parts of Fig. 2c were drawn by using a picture from Servier Medical Art. The body color was changed. Servier Medical Art by Servier is licensed under a Creative Commons Attribution 3.0 Unported License (https://creativecommons.org/licenses/by/3.0/). Source data are provided as a Source Data file.

We explored the spatial distribution of *IGF2* and *H19* expressions in 18 liver sections from 16 non-mosaic patients. In these patients, RNAscope analyses revealed that normal hepatocytes expressed both *IGF2* and *H19* known to originate from paternal and maternal alleles in the first years of life, respectively. In contrast, in 6 out of 8 mosaic patients demonstrating 11p15.5 cn-LOH (using WES or WGS), we identified typical benign expansions of hepatocytes displaying *IGF2* overexpression co-occurring with *H19* downregulation using singleplex or duplex *IGF2*/*H19* RNAscope assay (Fig. 2a; Supplementary Figs. 9 and 10). Beta-catenin immunostaining in these mosaic areas did not reveal a surrogate signal of β-catenin activation, neither with β-catenin overexpression nor its nuclear translocation. Moreover, these 11p15.5 locus altered regions did not show Glutamine synthetase (GS, encoded by *GLUL*) overexpression, in agreement with an absence of β-catenin activation in mosaic 11p15.5 altered livers. On the contrary, tumors altered for *CTNNB1* showed β-catenin and/or GS overexpression. In these liver sections, *IGF2* and *H19* expression were spatially heterogeneously distributed, with various proportions of *IGF2* overexpressing cells. This heterogeneity in patients was also retrieved in multi-sample analysis corresponding to various percentages of mosaic 11p15.5 altered cells assessed by WES/WGS sequencing or IC1/IC2 methylation analyses (Fig. 2b). For example, patient #3370 harbored a mosaic 11p15.5 cn-LOH in 33% of cells in the liver biopsy at diagnosis. At surgery, RNAscope in situ hybridization of *IGF2* and *H19* revealed the presence of 14% of mosaic cells in one FFPE non-tumor slide but not in the frozen sample (Fig. 2b, c). These results showed that spatial RNAscope duplex *IGF2*/*H19* hybridization assay can diagnose very heterogeneous expansions of mosaic 11p15.5 altered hepatocytes in FFPE liver sections. However, consistent with molecular results, no *IGF2* overexpression using RNAscope was observed in 2 mosaic 11p15.5 cn-LOH: a 3-year-old patient (#3383) with very low *IGF2* expression in bulk RNAseq and another patient (#3131) with 11p15.5 mosaic cn-LOH identified only in 3% of cells in the lung.

### 11p15.5 mosaic cells disrupt liver zonation architecture

**Fig. 3:**
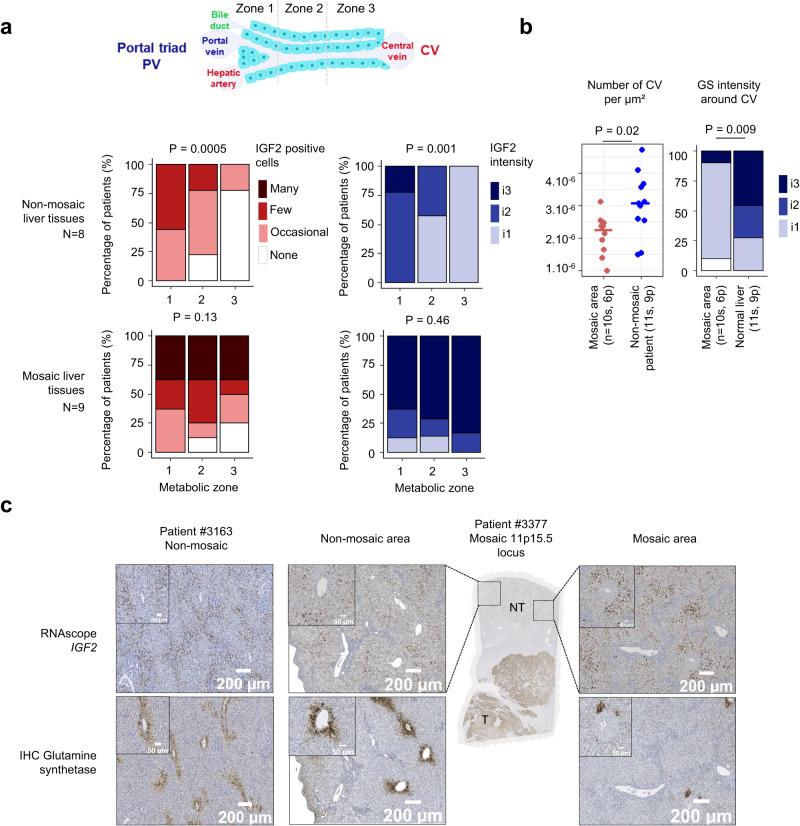
11p15.5 mosaic hepatocytes disrupt liver zonation architecture. **a** Frequence of *IGF2* positive cells (none to many) and *IGF2* intensity in metabolic zones (1 to 3) where i3 stands for highest intensity in 8 non-mosaic and 9 mosaic livers (11 and 10 analyzed slides respectively). Two-sided *χ*² for trend were performed. CV: central vein, PV: portal vein. **b** Number of central veins per µm² analyzed and glutamine synthetase staining intensity around CV in 8 non-mosaic and 9 mosaic livers (11 and 10 analyzed slides respectively). “s” indicates the number of slides analyzed and “*p*” the number of corresponding patients. Two-sided Wilcoxon and *χ*² for trend statistical tests were performed. **c** Representative RNAscope of *IGF2* and immunostainings of glutamine synthetase in paraffin-embedded liver sections from a non-mosaic patient (#3163), a non-mosaic and a mosaic area from a patient with 11p15.5 locus mosaic cn-LOH (#3377). All *IGF2* isoforms (P0 to P4 promoters) were considered in *IGF2* singleplex and duplex RNAscope assay. Two slides from patient #3377 and one slide from patient #3163 were analyzed. Scale bars, 50 µM and 200 µM. Source data are provided as a Source Data file.

To investigate the functional impact of 11p15.5 alteration on liver architecture, we explored liver zonation comparing mosaic expansions in 8 patients with 9 normal livers of patients at the same age (≤3.3 years old). In normal livers, *IGF2* expression gradually increased from zone 3 (perivenous area) to zone 1 (periportal area) both for the percentage of *IGF2* positive cells (*P* = 5.0 x 10^−4^) and *IGF2* expression intensity (*P* = 1.0 x 10^−3^) (Fig. 3a). Also, expression of glutamine synthetase (GS) was restricted around the central veins and spatially exclusive from *IGF2* expression. Similar observations were identified in bulk RNAseq expression data of 23 non-mosaic liver tissues: *IGF2* expression correlates with periportal markers (*PCK1*, *ASL*, *SDS*) and inversely correlates with perivenous markers (*GLUL*, *CYP1A2*, *CYP2E1*) (Supplementary Fig. 11a, b). In contrast, within the mosaic liver area, *IGF2* overexpression colonized periportal and perivenous areas as well, suggesting that mosaic clonal expansions alter the zonal architecture of the liver. This hypothesis was supported by the identification of frequent abnormalities in mosaic liver sections that exhibit incomplete GS zonation around central veins and/or the absence of central veins (Fig. 3b, c and Supplementary Fig. 11c). Overall, mosaic areas presented a lower number of central veins per µm^2^ (*P* = 0.02) and a GS staining around the central veins significantly less intense in comparison with normal livers (*P* = 0.009). This spatial alteration of the zonation in mosaic livers leads to a global lower perivenous metabolic program in bulk RNAseq compared to normal livers (*P* = 0.03, Supplementary Fig. 11b).

### Spatially resolved transcriptomic deregulation of 11p15.5 mosaic cells

**Fig. 4:**
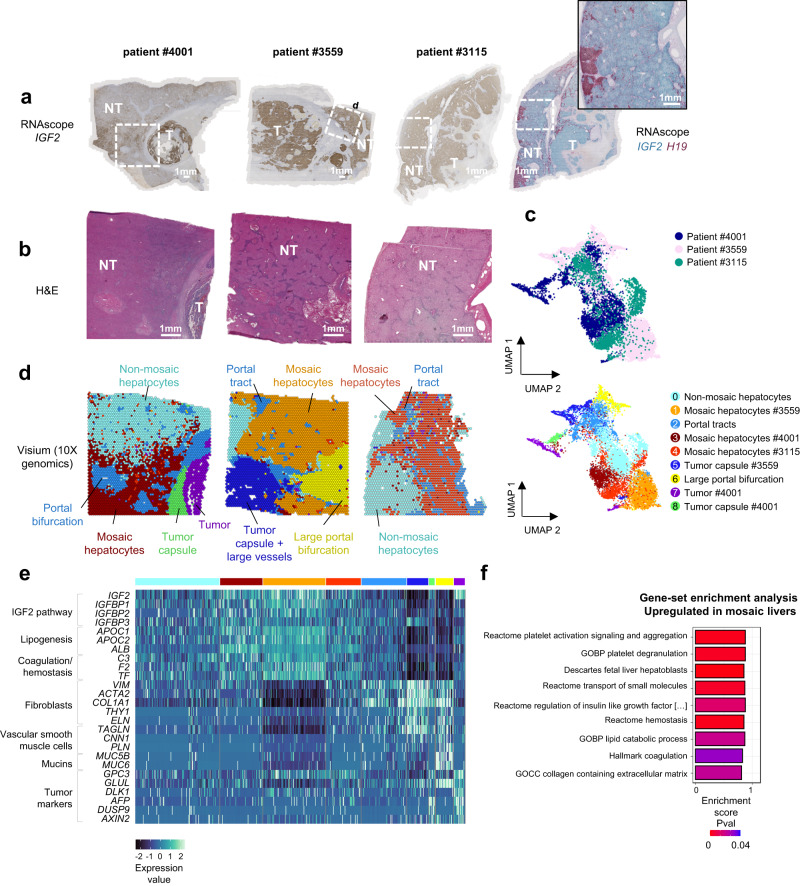
Spatial transcriptomics in patients #4001 #3559 and #3115 with mosaic cn-LOH at locus 11p15.5. **a** *IGF2* RNAscope in situ hybridization assay in 3 patients with mosaic cn-LOH. Three slides from patient #4001, 2 from patient #3559, and 3 from patient #3115 were analyzed. Scale bars, 1 mm. **b** Hematoxylin and eosin staining in FFPE sections from mosaic cn-LOH patients #4001, #3559, and #3115 realised during the spatial transcriptomics experiment. **c** UMAP representation of visium spatial transcriptomics (10X genomics) merged expression data from #4001, #3559, and #3115 samples and **d** spatial clusters visualization in FFPE slides. **e** Heatmap representation of differentially expressed genes between clusters. A two-sided Wilcoxon–Mann–Whitney test was performed. All *IGF2* isoforms were taken into consideration in *IGF2* spatial transcriptomics quantification. **f** Gene-set enrichment analysis between mosaic and non-mosaic hepatocytes from all 3 merged patients. Source data are provided as a Source Data file.

To investigate the transcriptomic deregulations resulting from 11p15.5 locus pre-malignant alteration in hepatocytes, we analyzed FFPE sections from 3 patients (#4001, #3559, and #3115) with 11p15.5 locus cn-LOH in the non-tumor liver using Visium spatial transcriptomics (10X genomics). Patients #4001, #3559, and #3115 carried 6%, 30%, and 58% of mosaicism in the non-tumor liver in WES or WGS, respectively. FFPE sections were selected based on Hematoxylin and Eosin staining and *IGF2*/*H19* RNAscope assays (Fig. 4a, b). Principal component analysis (PCA) and non-supervised clustering on the merged *Seurat* object (including the 3 patients) allowed identifying 9 robust cellular populations (Fig. 4c, d and Supplementary Fig. 12a). In each patient, two populations of hepatocytes spontaneously clustered separately and showed differential levels of *IGF2* expression. Overall, non-mosaic hepatocytes from all 3 patients clustered in a single group whereas mosaic hepatocytes with high *IGF2* expression corresponded to 3 specific clusters of each patient. Mosaic hepatocytes displayed high expression of *IGF2* pathway partners (*IGFBP1*, *IGFBP2*, *IGFBP3*) and genes involved in lipid metabolism (*APOC1*, *APOC2*) (Fig. 4e). Gene-set enrichment analysis between mosaic 11p15.5 cells from all 3 patients compared with non-mosaic cells revealed enrichment in fetal hepatoblast, platelet activation, transport of small molecules and lipid metabolism (Fig. 4f; Supplementary Data 5 and 6). Extracellular matrix was more abundant in the mosaic area in patients #4001 and #3115 but not in patients #3559. Of note, mosaic 11p15.5 areas did not show overexpression of Wnt/β-catenin target genes (*TBX3*, *GLUL*, *AXIN2*, *LGR5*, *LEF1*, Supplementary Fig. 13). Only *GPC3* progenitor marker gene identified in bulk RNAseq was overexpressed in mosaic hepatocytes in spatial transcriptomics analysis. *KIT*, *CD34*, *THY1,* and EPCAM were expressed in large vessel structures and tumor capsules from patients #4001 and #3559. This suggests that most of the progenitor signature detected in bulk RNAseq was due to the microenvironment (fibroblasts, vascular smooth muscle cells, endothelial cells) and not to the mosaic hepatocytes themselves. Tumor cells from patient #4001 were characterized by overexpression of *AFP*, *GPC3*, *DUSP9*, *DLK1, GLUL*, and *AXIN2*, a marker of Wnt/β-catenin pathway activation. In addition, we were able to identify different populations of tumor capsule overexpressing cancer-associated fibroblasts (CAFs) markers such as *FAP*, *VIM,* and *ACTA2* as well as hepatic stellate cells markers (*HGF*, *LRAT*, *RGS5*) (Supplementary Figs. 14 and 15). A cluster of portal veins and portal bifurcation showed high expression of portal (myo) fibroblast markers (*THY1*, *ELN*, *COL1A1*). A large portal bifurcation from patient #3559 was particularly enriched in liver vascular endothelial cells, fibroblasts, vascular smooth muscle cells (*TAGLN*, *CNN1*, *PLN*), and cholangiocytes (*KRT17*, *SPP1*). Of note, we were able to identify several hematopoiesis foci characterized by *HBB* and *GYPE* markers expression in the fibrotic area around large vessels (#3559, #4001, #3115) and in the tumor capsule (#4001) vascular area (Supplementary Fig. 14).

### Mosaic 11p15.5 alterations are identified in hepatocytes and cholangiocytes

**Fig. 5:**
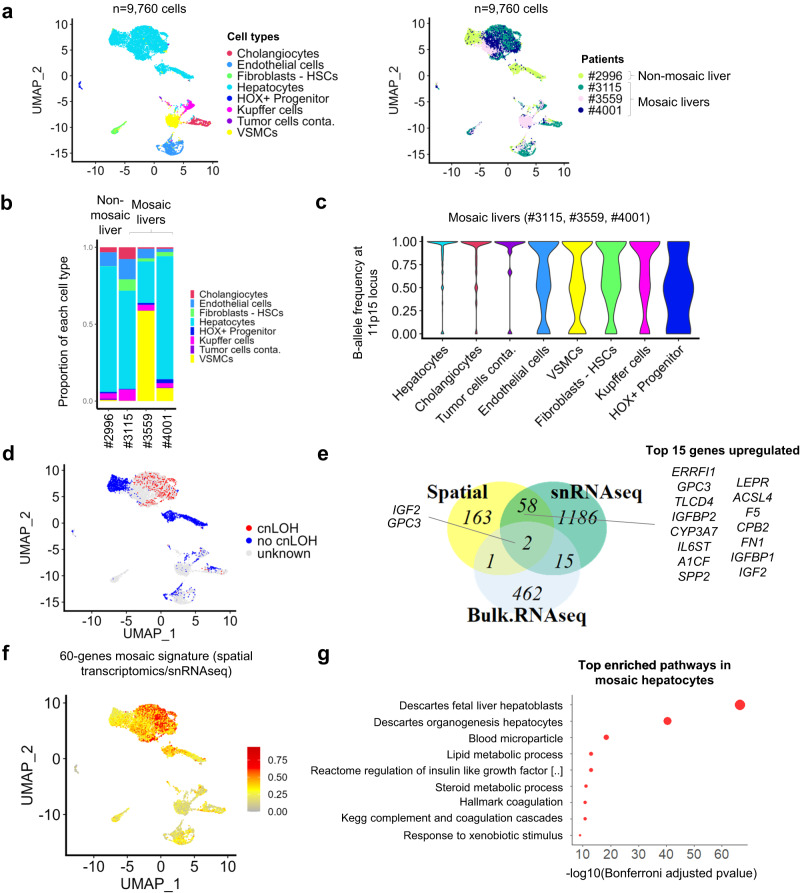
Single-nucleus RNAseq (snRNAseq) in 3 patients with mosaic cn-LOH at locus 11p15.5 and 1 patient without 11p15.5 mosaicism. **a** UMAP representation of snRNAseq (10X genomics) merged expression data from #4001, #3559, #3115 and #2996 samples. Left: annotation of the different cell types recovered. HSC: Hepatic stellate cells, VSMCs: Vascular smooth muscle cells. Right: patient of origin annotation. In total, 9760 cells were analyzed. **b** Proportion (0–1) of each cell population in all 4 snRNAseq samples. **c** In the 3 mosaic samples (#3115, #3559, #4001), B-allele frequency was assessed in 11p15.5 altered region specific of each patient in all cell types. The results indicate a cn-LOH in hepatocytes, cholangiocytes, and tumor cells. **d** UMAP representation of cn-LOH status per cell in the 4 patients. **e** Venn diagram representing the commonly upregulated genes in mosaic cells/livers using snRNAseq, spatial transcriptomics, and bulk RNAseq. The top 15 genes upregulated both in spatial transcriptomics and snRNAseq are highlighted. **f** Visualization of the 60-gene mosaic signature. **g** Functional enrichment (ToppGene suite) in mosaic hepatocytes based on 60 genes upregulated in snRNAseq and spatial transcriptomics. Source data are provided as a Source Data file.

To refine the transcriptomic signature of mosaic hepatocytes, we performed single-nucleus RNAseq (snRNAseq) of 3 mosaic livers with 11p15.5 cn-LOH (patients #4001, #3559 and #3115) and 1 non-mosaic liver (patient #2996) of similar age ranging from 2.6 to 17.4 months. After quality controls, we obtained 9,760 snRNAseq profiles (median 2778 UMIs and 1790 genes per nucleus, Fig. 5a and Supplementary Fig. 16a). Using an unsupervised clustering approach, we identified 8 cell populations: cholangiocytes, endothelial cells, fibroblasts/hepatic stellate cells, hepatocytes, Kupffer cells, vascular smooth muscle cells (VSMCs), a population of progenitor cells expressing high levels of *HOX* genes and few tumor contaminating cells (Fig. 5a; Supplementary Fig. 16b and Supplementary Data 7). Mosaic livers displayed higher proportions of VSMCs and fibroblasts/hepatic stellate cells (HSCs) which confirms the observation made in bulk RNAseq (Fig. 5b). In mosaic patients, high B-allele frequency (BAF) due to 11p15.5 cn-LOH was found in hepatocytes, cholangiocytes and contaminating tumor cells suggesting that the 11p15.5 alteration occurred in a hepatobiliary progenitor cell (Fig. 5c, d). Differential expression analysis of snRNAseq hepatocytes with cn-LOH identified a signature of 2,268 genes (1261 upregulated, 1007 downregulated) compared to hepatocytes without cn-LOH (Supplementary Data 8). Comparison of this snRNAseq signature with the spatial transcriptomics mosaic and bulk RNAseq revealed a high expression of *IGF2* and *GPC3* common to the 3 methods (Fig. 5e). We also found 58 other commonly upregulated genes in both snRNAseq and spatial transcriptomics techniques (Fig. 5f). Among them, we found an upregulation of *IGF2* partner genes (*IGFBP1*, *IGFBP2*), genes related to coagulation/thrombosis (*FN1*, *CPB2*, *F2*, *TF*, *F9*, *PROS1*, *AGT*), and lipid metabolic process (*ACSL4*, *AADAC*, *LIPC*, *PTGR1*, *LONP2*, *SOCS3*, *SCP2*, *GC*, *APOC1*, *APOC2*, *IL6ST*, *PRDX6*, *SLC27A2*) that highlight mosaic hepatocytes (Fig. 5f, g; Supplementary Fig. 16c, d and Supplementary Data 9).

### 11p15.5 locus and β-catenin cooperation in HB tumorigenesis

**Fig. 6:**
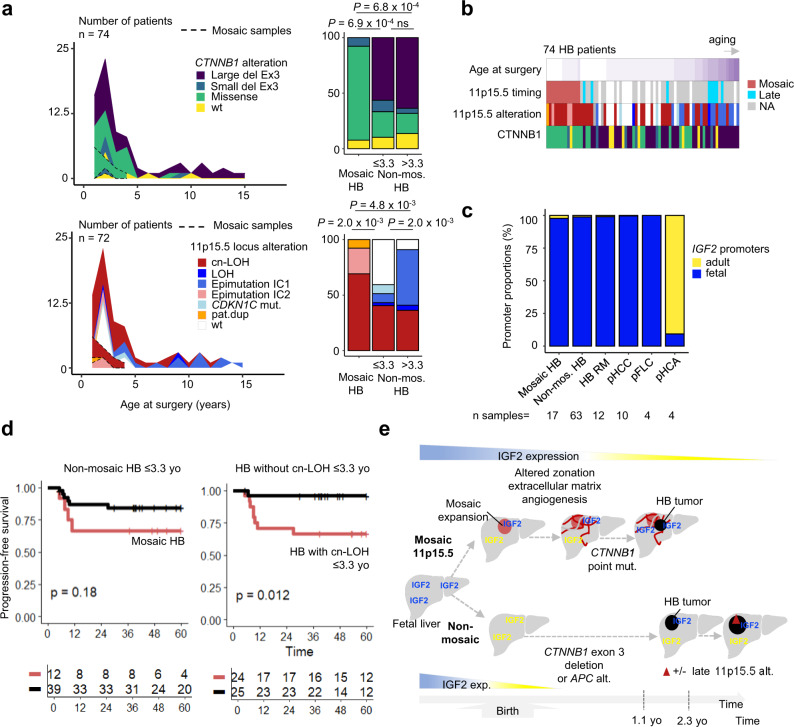
β-catenin and 11p15.5 locus oncogenic cooperation in mosaic and non-mosaic HB tumors. **a** Evolution of β-catenin and 11p15.5 locus alteration spectra with age at surgery in 74 and 72 patients, respectively. One sample per patient was represented. A two-sided *χ*² test was performed. **b** Heatmap representation of *CTNNB1* and 11p15.5 locus alterations in 74 HB tumors. **c** Proportions of fetal and adult *IGF2* promoter usage in 110 pediatric liver tumors. HB RM: hepatoblastoma relapse or metastasis. **d** First Kaplan–Meier curve indicates progression-free survival in mosaic (*n* = 12) and non-mosaic patients under 3.3 years of age (*n* = 39). The second Kaplan–Meier curve displays progression-free survival in patients with HB with cn-LOH (*n* = 24) or without (*n* = 25) under 3.3 years of age. Log-rank statistical test was performed. **e** Scheme representing different routes for HB tumorigenesis. HB can develop from 11p15.5 mosaic cells or directly in isolation through β-catenin alteration. Mos.: mosaic, non-mos.: non-mosaic, pat.dup: locus 11p15.5 paternal duplication, yo: years old. alt: alteration, mut. mutation, cn-LOH: copy-neutral LOH, exp.: expression. Source data are provided as a Source Data file.

Finally, we analyzed 123 tumor samples from 74 HB patients (Supplementary Fig. 2). We identified *CTNNB1* and 11p15.5 locus alterations in at least one HB sample in 89% (66/74) and 76% (55/72) of the patients, respectively. Of note, we found a damaging *APC* mutation in 7 patients (6 constitutional and 1 somatic alteration) and 1 patient displayed *AXIN1* germline alteration, exclusive from *CTNNB1* alterations. To investigate the mechanism of cooperation of these two frequent alterations, we first compared *CTNNB1* and 11p15.5 spectra of alterations in the tumors occurring in patients with or without 11p15.5 mosaic in their corresponding non-tumor liver. HB derived from mosaic liver were highly enriched in *CTNNB1* point mutations, mostly located at the β-TRCP binding domain (p.D32, p.S33, p.G34, p.S37, 8 cases) or at p.T41 (3 cases), compared to non-mosaic HB at the same age (*P* = 6.9 x 10^−4^). In contrast, non-mosaic HB displayed frequent *CTNNB1* large in-frame exon 3 deletions, associated with IC1 epimutation in the oldest patients (*P* = 2.0 x 10^−3^, Fig. 6a).

Then, we addressed the question of the timing of the occurrence of 11p15.5 alterations in the liver. In 12/13 patients with 11p15.5 mosaic alterations in the non-tumor liver tissues, the exact same alteration was identified in the corresponding HB tumor. However, in these mosaic non-tumor liver tissues, we neither identify *CTNNB1* oncogenic mutation nor an alteration in other cancer driver genes. In contrast, *CTNNB1* mutations were identified together with the 11p15.5 alterations in their corresponding tumor counterparts in 12 out of 13 patients. This observation demonstrated that in all but one HB developed in the context of mosaic benign lesions, 11p15.5 alteration preceded the occurrence of *CTNNB1* alteration. The remaining mosaic patient (#3608) displayed IC1 and IC2 epimutations in the liver and a cn-LOH in the tumor.

Using phylogenic reconstruction from multiple samples in 25 patients, in 10 of them, we identified late 11p15.5 alterations (4 IC1 epimutations, 4 cn-LOH, 2 patients with late cn-LOH and IC1 epimutation in 2 different samples) occurring after the onset of *CTNNB1* alteration (*n* = 9), and one after *APC* germline alteration (Fig. 6b). Clinical and molecular features of HB with late 11p15.5 alteration identified frequent *TERT* alteration (*P* = 0.006) and a tendency to be older (5.9 years old, *P* = 0.23, Supplementary Table 1. These results indicate that 11p15.5 locus alteration can both play a role in predisposition and HB progression.

We then analyzed the relationship between 11p15.5 locus alterations in HB and published transcriptomic classifications^6,10,14,22^. Overall, 11p15.5 alterations were enriched in C2^10^, C2A/C2B^22^, proliferative/mesenchymal^14^, and mesenchymal/liver progenitor^6^ transcriptomic subgroups (Supplementary Fig. 17).

Strikingly, all malignant pediatric liver tumors (HB, pHCC, and pFLC) showed massive usage of *IGF2* fetal promoters, which were hypomethylated (Fig. 6c and Supplementary Fig. 18a). In contrast, hepatocellular adenomas expressed mostly adult *IGF2* transcripts. Among HB without mosaicism, tumors with 11p15.5 alteration showed a higher *IGF2* expression (*P* = 0.01) and a lower *H19* and *CDKN1C* tumor suppressor expression (*P* = 5.3 x 10^−8^ and *P* = 4.0 x 10^−3^, respectively). Overall, *IGF2* expression in tumor and non-tumor pediatric liver tissues was regulated both through promoter usage and parental imprinting. Of note, in pFLC despite the usage of fetal promoters, all harbored an under-expression of *IGF2*, including in one case with an 11p15.5 cn-LOH (Fig. 6c and Supplementary Fig. 18b). This suggests that the pro-oncogenic role of *IGF2* in HB and pHCC are not present in pFLC.

### Histological, molecular, and clinical features of mosaic HB

Among children with HB before 3 years old, 11p15.5 mosaic patients tend to have lower progression-free survival (PFS; *P* = 0.18, Fig. 6d) although they did not exhibit a significant difference in tumor size, PRETEXT stage, number of nodules, or presence of metastasis at diagnosis (Supplementary Table 1). Interestingly, 11p15.5 cn-LOH, which is the major mechanism of mosaicism, is associated with poor PFS in young patients (*P* = 0.01, Fig. 6d), indicating that the presence of a cn-LOH rather than mosaicism is the most predictive of a poor prognosis.

## Discussion

There is growing evidence that driver mutations present in cancers can occur during embryogenesis^23,24^. In line with these findings, we identified in this study mosaic clonal pre-malignant expansions of hepatocytes with 11p15.5 locus alteration in 17% of children with HB. In all these patients, HB tumors derived from the malignant transformation of the mosaic clones with 11p15.5 alteration with additional *CTNNB1* oncogenic mutation in the tumor cells (Fig. 6e). Preneoplastic 11p15.5 alterations were exclusively identified in children with HB but not with other types of liver cancer (pHCC, pHCA or pFLC). However, the overall 11p15.5 alteration frequency may be underestimated considering the high degree of spatial and longitudinal heterogeneity of mosaic areas and additional samples in pediatric and adult liver tissues remain to be tested.

In our study, we discovered 11p15.5 mosaic alterations in 12 of the 13 children in the absence of clinical Beckwith-Wiedemann syndrome. Only one child demonstrated clinical BWS and 11p15.5 altered blood cells. Also, HB developed from mosaic 11p15.5 altered clones were identified in children particularly young (median=1.01-year-old), similarly to the BWS patient, suggesting that liver tumorigenesis could initiate early in life, probably before birth. Accordingly, 11p15.5 mosaic alterations were identified in both hepatocytes and cholangiocytes suggesting that the genomic defect occurred at the hepatobiliary progenitor stage. Recently, 11p15.5 mosaic alterations in kidneys of children with nephroblastoma have been described in ~37% of patients^25^. Most of these patients were not associated with clinical BWS and/or alterations in blood cells. Genetic alterations that occur during embryonic development have the particularity of altering a variable fraction of the organs depending on the developmental stage at which the alteration occurs. In Wilms tumors, clonal nephrogenesis was associated with bilateral tumors suggesting that mosaic alteration occurred before the diversion of left and right kidney primordia. In the liver, the timing of the occurrence of 11p15.5 alterations during embryogenesis remains to be estimated.

The mechanism of 11p15.5 alterations varies according to organs and pediatric tumor type. Indeed, 11p15.5 cn-LOH is predominant in preneoplastic mosaic livers, IC1 hypermethylation in pre-malignant mosaic kidney^25^ whereas in BWS the major alteration is IC2 epimutation^26^. This observation suggests that cn-LOH could predispose more to the malignant transformation than other alterations of the 11p15.5 locus in the liver. Indeed, 11p15.5 cn-LOH leads to a decrease in tumor suppressor *CDKN1C* gene expression in addition to the oncogenic expression of *IGF2*. We can hypothesize that both events are necessary to initiate the cell toward a malignant transformation in the liver.

Functional consequences of 11p15.5 alterations are important for understanding early mechanisms of malignant transformation in preneoplastic clones. Copy-neutral LOH at 11p15.5 locus leads to an overexpression of *IGF2* in mosaic livers. Using spatial transcriptomics analyses, we identified that many overexpressed genes in mosaic livers were expressed by cells from vascular structures rich in (myo)fibroblasts, hepatic stellate cells, and vascular endothelial cells. Moreover, mosaic hepatocytes showed an upregulation of coagulation and platelet signaling pathways. Therefore, mosaic 11p15.5 alteration could provide a pro-angiogenic niche through IGF2 since it has been proposed as a pro-angiogenic growth factor during late placental development controlling feto-placental vascularization and trophoblast morphogenesis^27^. In this line, BWS patients can exhibit placentomegaly caused by hypervascularization and hyperplasia^28,29^. In addition, a recent genomic study identified recurrent cn-LOH of 11p15.5 locus in the placenta (2/42 ~ 5%) indicating that these alterations can be relatively frequent in placenta^30^ and may confer a selective advantage for placental growth. Moreover, adult HCCs with high IGF2 expression up-regulate vascular endothelial growth factor A (VEGFA) targets, hepatic stellate cells, and epithelial-to-mesenchymal transition signatures^31^. Besides vascular abnormalities, mosaic 11p15.5 hepatocytes showed overexpression of protein synthesis and lipogenesis related to INSR-A binding and insulin pathway activation. However, we did not observe cell cycle deregulations in our mosaic livers as it was reported in HB associated with BWS^32^.

In normal livers, *IGF2* was mostly expressed in periportal areas (zone 1) surrounding liver lobules whereas zonation was disrupted in mosaic livers with *IGF2* high expression in all areas. Mosaic livers also presented an alteration of the zonal architecture of the liver possibly due to the clonal expansion of 11p15.5 altered hepatocytes that prevent their zonal specification and could result in metabolic perturbations. Since no mosaic 11p15.5 altered hepatocytes have been identified in the liver of children older than 3.3 years we can hypothesize that these mosaic hepatocytes could be functionally counter-selected and clonal expansions could regress to ensure a full metabolic function of the liver in the first few years of life. Recently, mosaic hotspot mutations in *FOXO1*, a major transcription factor of the insulin response, have been reported in adult patients with liver diseases^33^. Interestingly, in children and adults, major actors of the insulin signaling pathway (*IGF2* and *FOXO1*) were identified as mosaic alterations in the liver. Their consequences on the homeostasis of the liver tissue would be important to compare.

Mosaic hepatocytes are benign suggesting that 11p15.5 alteration is not sufficient to ensure a malignant transformation but requires ß-catenin activation for HB development. This co-occurrence argues for cooperation between the two pathways towards oncogenesis. Analysis of *CTNNB1* and 11p15.5 locus alteration spectra revealed that mosaic HB were enriched in β-catenin point mutations whereas non-mosaic HB presented large β-catenin deletions, associated with IC1 epimutations in the oldest patients. Therefore, it suggests different natural histories leading to HB development: HB can arise either in an 11p15.5 predisposing context (cn-LOH or epimutation IC2) followed by a *CTNNB1* point mutation or directly through *CTNNB1* large deletion and synchronous or late 11p15.5 cn-LOH/IC1 epimutation. Interestingly, in nephroblastoma, 11p15.5 cn-LOH and mosaic IC1 hypermethylation were almost mutually exclusive indicating two pathways towards oncogenesis both utilizing 11p15.5 genes dysregulation as a driver.

Copy-neutral-LOH alteration at 11p15.5 locus is associated with low PFS in children before 3 years old, therefore we can hypothesize that 11p15.5 alterations occurring early in life could be associated with a poor prognosis due to cell plasticity as previously described in HB^6^. Since cn-LOH is the major mechanism of mosaic 11p15.5 alterations we need to better understand if HB patients would benefit from a systematic and sensitive diagnosis of mosaicism and from a specific follow-up. Loss of parental imprinting in mosaic livers can be assessed using targeted methylation kit (MS-MLPA) and *IGF2*/*H19* gene expression changes can be detected directly in FFPE slides using in situ hybridization assay. This would also allow for better characterization of these pre-malignant clonal expansions for the benefit of the patient.

In conclusion, mosaic pre-malignant expansion of clonal hepatocytes harboring 11p15.5 alteration determines a specific sub-group of young children with HB more at risk of early relapse and metastasis after the first line of treatment for those with cn-LOH. Future prospective follow-up of patients with mosaic 11p15.5 alteration would precisely characterize putative therapeutic consequences of these genomic alterations to adapt the care and surveillance of the patients.

## Methods

The study was approved by the local Ethics Committee (CCPRB Paris Saint-Louis) and written informed consent was obtained from all participants or their parents or legally authorized individuals.

### Clinical samples

A cohort of 131 patients including 115 patients with pediatric liver cancers (77 HB, 12 pHCC, 9 pFLC, and 17 pHCA) were collected from different French hospitals. In addition, 16 fetal liver samples from abortions were collected by the Centre de Ressources Biologiques (CRB) Bordeaux, in agreement with the institutional review board committee (approval number 2010-A00498-31). Frozen tissue samples were immediately flash-frozen in liquid nitrogen and stored at −80 °C. Among non-tumor HB samples, 7 were resected prior (1 mosaic) and 70 after chemotherapy (12 mosaic) whereas 18 HB tumor samples were resected before chemotherapy and 105 post-chemotherapy. Most HB were sporadic but 6 developed with Familial adenomatous polyposis (FAP), 1 with BWS, 1 with Simpson Golabi Behmel syndrome, and one in the context of Duchenne myopathy. All pFLC occurred in the absence of underlying etiology. Among pediatric HCC, 4 developed with Progressive familial intrahepatic cholestatis (PFIC), 4 with mitochondrial cytopathy, 2 patients developed in the context of Tyrosinemia, and 1 with hepatoportal sclerosis. Two pHCA developed with underlying biliary atresia, 3 with congenital portocaval shunt, 1 with glycogenosis, 1 with Alagille syndrome, 1 with sickle cell disease and 1 associated with anabolic steroids use. A detailed description is provided in Supplementary Data 1.

### Whole-genome and whole-exome sequencing

Tumor and non-tumor DNA were extracted using either the Maxwell 16 tissue DNA purification kit (Promega, ref # AS1030) or the AllPrep DNA/RNA/miRNA Universal kit (Qiagen, ref #80224). WGS from 116 samples (51 non-tumor and 65 tumor samples) were performed at the Centre National de Recherche en Génomique Humaine (CNRGH, Evry, France) on an Illumina HiSeqX5 with paired-end reads and an average depth of 30× for non-tumor samples and 90× for tumors. Among them, 3 non-tumor samples were not previously published. Reads were then aligned on hg19 reference genome using the Burrows-Wheeler Alignment tool (BWA)^34^. Picard tools (http://broadinstitute.github.io/picard/) were used to remove PCR duplicates, GATK tool (https://gatk.broadinstitute.org/hc/en-us) for local indel realignment and cgpBattenberg (https://github.com/cancerit/cgpBattenberg) algorithm to reconstruct copy-number profiles. Whole-exome sequencing from 97 samples (44 non-tumor and 53 tumor samples, 6 not previously published) was performed by IntegraGen SA (Evry) on an Illumina HiSeq4000 (paired-end 75 bp reads) or Illumina NovaSeq (paired-end 100 bp reads). The average sequencing depth was 100x for tumors and 65x for non-tumor samples. Reads were then aligned on the hg38 genome reference. When non-tumor and tumor samples were not sequenced in WGS or WES, *CTNNB1,* and *TERT* alterations were screened in targeted Sanger sequencing^35^. *CTNNB1* deletions were considered as “small deletion exon 3” when they did not imply a complete deletion of exon 3 sometimes encompassing the beginning of exon 4.

### Phylogenetic reconstruction

Phylogenetic trees were constructed for patients with multiple tumor samples as described in Hirsch et al. ^6^. Briefly, the cancer cell fraction (CCF) for each mutation was estimated using the Palimpsest tool. Then, we used a Dirichlet process to find groups of mutations with similar CCF corresponding to the same branch of the phylogenetic tree.

### RNA sequencing

RNAseq was performed on 7 fetal livers, 33 non-tumor counterparts of HB, 4 counterparts of pHCC, 3 of pFLC, 3 of pHCA as well as 117 tumor samples of which 45 were not previously published. RNAseq was performed as described in Hirsch et al.^6^. Briefly, TopHat2 was used to align full Fastq files with human genome hg38. HTSeq was used to quantify reads aligned on each gene, and DeSeq2 to normalize expression data through variance stabilization.

### Detection and quantification of mosaic 11p15.5 altered cells

We identified 13/77 samples with 11p15.5 locus mosaicism using a multi-techniques approach. First, using WGS and WES, for each tumor with a copy-number alteration at locus 11p15.5 (cn-LOH or paternal duplication), we searched for the presence of the same alteration in non-tumor counterpart. For this purpose, we computed the *BAF* for common single-nucleotide polymorphisms (SNPs) in non-tumor tissue (*BAF*_NT_) and in the tumor (*BAF*_T_). We then performed a binomial test for SNPs with the major B-allele in the tumor (*BAF*_T_ > 0.5) and those retained in the non-tumor *BAF*_*N*T_ > 0.5. The percentage of 11p15.5 altered cells was calculated using the formulae:

1 $$\%\,{{{{{\rm{cells}}}}}}\,{{{{{\rm{altered}}}}}}=100\times \frac{2\times {{GAF}}_{{NT}}-1}{{{GAF}}_{{NT}}\times \left(2-{N}_{{maj}}-{N}_{\min }\right)+{N}_{{maj}}-1}$$

where GAF_NT_ is the median BAF of the gained alleles at 11p15.5 locus in the non-tumor sample, and *N*_min_ and *N*_maj_ are, respectively, the number of minor and major alleles at this locus. In the specific case of cn-LOH, since *N*_min_ = 0 and *N*_maj_ = 2 we have:

2 $$\%\,{{{{{\rm{cells}}}}}}\,{{{{{\rm{altered}}}}}}=100\,{{{{{\rm{x}}}}}}\,(2\,{{{{{\rm{x}}}}}}GA{F}_{NT}-1)$$

Then, using RRBS and/or MS-MLPA methylation values, the percentage of mosaic 11p15.5 cells in non-tumor livers was calculated with the following formulae:

3 $$\%{{{{{\rm{cells\; altered}}}}}}=100\times \frac{{IC}1-{IC}2}{{IC}1+{IC}2}$$

When FFPE slides were available, non-tumor liver areas with high *IGF2* and low *H19* expression in singleplex or duplex RNAscope stainings were annotated as mosaic 11p15.5 areas (Mosaic area_*NT*_) with QuPath software. The total non-tumor slide area was determined with QuPath software as well (Total slide area_*NT*_). Then, the percentage of mosaic cells was determined as follows:

4 $$\%\, {{{{{\rm{cells}}}}}}\; {{{{{\rm{altered}}}}}}=100\times \frac{{{{{{{{\mathrm{Mosaic}}}}}}}\,{{{{{{\mathrm{area}}}}}}}}_{{NT}}\,}{{{{{{{{\mathrm{Total}}}}}}}\,{{{{{{\mathrm{slide}}}}}}}\,{{{{{{\mathrm{area}}}}}}}}_{{NT}}\,}$$

### Methylation-specific multiplex ligation-dependent probe amplification (MS-MLPA)

To assess IC1 and IC2 methylation levels, we used MS-MLPA ME030-C3 BWS/RSS kit (MRC-Holland) in which ten probes were methylation-specific (4 IC1, 4 IC2, 2 targeting *IGF2* promoter methylation). We excluded one probe from the analysis targeting IC1 (H19.11.001.976583) because its distribution did not discriminate between samples with and without gain of methylation IC1 (GOM IC1). To determine tumors with GOM IC1 and/or loss of methylation IC2 (LOM IC2), we used the *k*-means clustering method. Tumor samples were annotated with GOM IC1 (respectively LOM IC2) when at least 2/3 (respectively 2/4) probes were above the threshold.

### Reduced representation bisulfite sequencing (RRBS)

*IGF2* promoter methylation was assessed using RRBS methylation data from 17 non-tumor samples and 87 tumor samples. RRBS was performed with 100 ng of genomic DNA from each sample at IntegraGen (Evry) according to Gu and colleagues’ protocol^36^. We used BS_Seeker2 to perform alignment on the hg38 genome version. *IGF2* promoters (adult promoter P1 and fetal promoters P2-P3-P4) were defined as follows: first, we displayed on the IGV software the distribution of CpG sites detected with RRBS (that are, the CpG sites detected in at least one of the 110 samples from the RRBS cohort). We then defined the promoters so that they contained CpG clusters in the neighborhood of the beginning of the corresponding exon and had a size of around 1000 bp. P1–P4 promoter coordinates (hg19) are available in Supplementary Table 2.

For each promoter, we removed the CpG sites located in RRBS-project-biased regions (since the RRBS cohort comprises 2 projects performed separately) before computing the mean methylation level for each promoter, defined as the ratio between the number of methylated cytosines and the total number of methylated and unmethylated cytosines.

In order to assess the methylation status of locus 11p15.5 imprinting centers, for each sample we calculated the mean methylation of IC1 (chr11:1998745–2003509, hg38) and IC2 (chr11:2697587–2700983, hg38). Samples with epimutation IC1 and IC2 were then identified using the *k*-means clustering method.

### *IGF2* isoforms quantification

*IGF2* transcripts from promoters P0-P4 were quantified using RNAseq data. For each sample, the maximum depth of junction in each category (P0-P1/P2/P3/P4) was extracted. Each value was normalized by the number of reads from “universal” junctions, defined as junctions shared by all transcripts. Finally, the number of junctions was converted into a proportion of promoter usage.

### In situ hybridization in FFPE slides

*IGF2* and *H19* singleplex assays were performed using RNAscope 2.5 HD Detection Reagent BROWN kit (cat. #322300) or RED kit (cat. #322350) according to the manufacturer’s protocol. Duplex hybridization of *IGF2* probe (cat. #594361) and *H19* probe (cat. #400771-C2) were performed using chromogenic RNAscope 2.5 HD Duplex reagent kit (cat. #322430). RNAscope analyses include all *IGF2* isoforms. Indeed, we used ~20 probes (ZZ pairs) targeting *IGF2* transcript variant 1, mRNA from nucleotide 692 to 2,021. The region targeted in IGF2 is shared by all isoforms as shown in the graphical representation in Supplementary Fig. 19. In all RNAscope assays, 5 µM FFPE sections underwent target retrieval for 15 min in target retrieval reagent >98 °C and a 20-min protease digestion at 40 °C.

### Immunohistochemistry (IHC)

Immunostainings of β-catenin (BD Biosciences, Mouse IgG1, clone 14 ref #610154, 1/200) and glutamine synthetase (BD Biosciences, Mouse IgG2a, clone 6, ref #610517, 1/500) proteins were performed using DAKO Autostainer AS48L. Antigen retrieval was done at pH6 and pH9 for glutamine synthetase and β-catenin stainings respectively.

### Pathological reviewing

Slides were reviewed by a consensus of 4 expert pathologists (CG, MF, BG, GM). In the non-tumor liver, *IGF2* intensity was graded from i1 (lowest) to i3 (highest) intensity in each metabolic zone (1 to 3). Accordingly, the number of *IGF2-positive* cells was ranked from no *IGF2-positive* cells to many *IGF2-positive* cells. To determine the impact of mosaic 11p15.5 pre-malignant expansions on liver zonation, glutamine synthetase (GS) intensity in mosaic and non-mosaic areas (see §*Detection and quantification of mosaic 11p15.5 altered cells*) was quantified using QuPath software^37^. Finally, the number of central veins was manually counted in mosaic and non-mosaic areas. In tumor slides, histological components were recorded according to the consensus classification^38^ as follows: fetal, embryonal, mesenchymal, small-cell undifferentiated, cholangioblastic.

### Spatial transcriptomics

Spatial transcriptomics was performed according to 10x genomics Visium protocol with prepared 5 μM FFPE sections of patients #3559, #4001, and #3115. Sequencing data from Visium slides were processed by spaceranger v1.3.1 using mkfastq and count spaceranger commands. All count matrix and image data were individually analyzed by R package *Seurat* v.4.0.1. Each data set was normalized using SCTransform Seurat function. One slide (#4001) showed a small tissue detachment on the top-right corner with low counts. Therefore, spots with less than 1500 counts for this patient were not included in the analysis. To analyze the slides together, the #4001, #3559 and #3115 datasets were merged using the Seurat merge function.

Dimensionality reduction was performed on merged data using the principal component analysis RunPCA function and the 40 first principal components were used in UMAP (Uniform Manifold Approximation and Projection) representations. In order to determine clusters, FindNeighbors and FindClusters Seurat functions were used with a resolution value of 0.40. Population clusters were assigned with unsupervised clustering. To check for the clustering robustness, we used *clustree* function (Supplementary Fig. 12a). To identify a putative batch effect, we used *harmony* package v0.1.1 using *runHarmony* R function and performed non-supervised clustering on the merged object. We obtained very similar results with 9 clusters (0-8) at a resolution of 0.3 (Supplementary Fig. 12b). PrepSCTFindMarkers function was used to remove batch effect before differential expression analysis between clusters. Differentially expressed markers between groups were detected using FindMarkers function and visualized with SpatialFeaturePlot function. All *IGF2* isoforms were considered with an *IGF2* probe as described in Supplementary Fig. 19. Gene-set enrichment analysis (GSEA) was performed on ranked differentially expressed markers using *fgsea* R package v.1.20.0.

### Single-nucleus RNAseq experiment

Four samples (#3115, #3559, #4001, and #2996) were sequenced in snRNAseq (10x genomics) according to the manufacturer’s protocol. Briefly, nuclei isolation from frozen tissues was done as described in Slyper et. al Nature Medecine 2020^39^, using EZ Lysis buffer workflow with slight modifications. Briefly, tissue samples, thawed in a drop of 200 µl of PBS, were cut, into pieces <0.5 cm. For the tissues in OCT, a few minutes were necessary to separate the tissues from the OCT and then to manage them like the others. Approximately 35 mg +/− 10 mg of tissue was poured into a glass Dounce tissue grinder (Sigma, cat. no. D8938) and homogenized. The tissue was homogenized 25 times with pestle A and 25 times with pestle B in 1.5 ml of ice-cold nuclei EZ lysis buffer. The sample was then incubated on ice for 5 min, with an additional 3 ml of cold EZ lysis buffer. Nuclei were centrifuged at 500 × *g* for 5 min at 4 °C, washed with 5 ml ice-cold EZ lysis buffer, and incubated on ice for 5 min.

After centrifugation, the nucleus pellet was washed with 5 ml of Nuclei Suspension Wash buffer (PBS, 0.1% BSA, 200 u/ml RNase Inhibitor (Sigma). Then the pellet was resuspended in a volume of Nuclei Suspension buffer (PBS, 1% BSA, 200 u/ml RNase Inhibitor (Sigma)). and filtered through a 70 µm MACS SmartStrainers (Miltenyibiotec 130-098-462) and then a 35 µM Cell Strainer (Corning 352235). Nuclei were counted under a microscope using C-chip disposable hemocytometer. If possible, a final concentration between 700 and 1200 nuclei per µl is used for the Chromium Next GEM Single Cell 3’ kit. The CG000315_Chromium NextGEM Single Cell 3’ v3.1 Dual Index User Guide_RevE (10X genomics provider) was followed for GEM generation, Gene Expression library construction. Nuclei were loaded on the Chromium Controller to target 5000 recovered nuclei.

After qualitative control on Fragment Analyzer and quantitative control by qPCR, the libraries are finally sequenced in 100pb*100pb on Novseq6000. Image analysis and base calling are performed using Illumina Real Time Analysis with default parameters.

### Detection of 11p15.5 copy-neutral LOH in single-nucleus data

We used germline heterozygous single-nucleotide polymorphisms (SNPs) to detect 11p15.5 copy-neutral LOH in snRNAseq data. For each sample, we used the distribution of B-Allele Frequency (BAF) in WGS data of matched non-tumor and tumor samples in the region of cn-LOH. BAF values in the non-tumor samples were used to filter out germline homozygous SNPs and only keep heterozygous SNPs. BAF values in the tumor, where there is a strong disequilibrium due to the cn-LOH, were used to assign for each SNP the paternal (PAT, duplicated) and maternal (MAT, lost) allele. We then used screadcounts (version 1.3.1, options –C STARsolo –b cell IDs --umicount CellRanger)^40^ to count the number of PAT and MAT alleles for each SNP in each cell. We summed the number of PAT and MAT reads in each cell, and computed the BAF per cell, defined by the ratio PAT/(PAT + MAT). Focusing on cells with at least 8 informative reads, we considered a cell as harboring the cn-LOH if the BAF was ≥85% and not harboring the cn-LOH if the BAF was ≤60%, while other cells were considered unknown. Finally, all cells from sample #2996, which did not harbor any cn-LOH in its tumor, were considered as ‘no cn-LOH’.

### Bioinformatics analyses of snRNAseq data

All data were analyzed using *Seurat* package v4.3.0^41^. Cells with more than 1000 genes detected and less than 5% of mitochondrial genes were kept for analysis. For each sample, a cluster of cells with high mitochondrial genes and high ambient RNA contamination was removed for further analysis. After quality controls, we retrieved 4136 cells for #3115, 1388 cells for #4001, 2275 cells for #2996, and 1961 cells for #3559 (see Supplementary Fig. 16a). Gene expression normalization was performed separately in each sample using *SCTransform* function. Then, all 4 samples were combined using *Seurat* merge function. Variable features of the merged object were calculated using *SelectIntegrationFeatures* function and dimension reduction was performed using *RunPCA* with 50 components. The top 30 components were used for UMAP representations, and we set a seed to 1234. Unsupervised clustering was performed using *FindNeighbors* and *FindClusters* functions and a resolution of 0.2. We then assigned cell populations using the top differentially expressed genes in each cluster (see Supplementary Data 7) and manual annotation with curated markers from the literature. We annotated each cell with the corresponding cn-LOH status according to paragraph method *§11p15.5 cn-LOH status assignment in snRNAseq*. We compared gene expression between hepatocytes with and without cn-LOH with *FindMarkers* function with a minimum log Fold change of 0.1 and an expression in at least 25 percent of the cells in either population. Genes were considered as differentially expressed when the adjusted *p*-value was below 0.01. Functional enrichment was performed using ToppGene suite and the 60 commonly deregulated in snRNAseq and spatial transcriptomics.

### Statistics and reproducibility

All statistical analyses were performed using R v.4.2.1. Two-tailed Mann–Whitney Wilcoxon tests were performed to compare two groups. When the population was normally distributed, Student *t*-test was performed to compare two conditions. Kaplan–Meier survival curves were compared with the log-rank test. No statistical method was used to predetermine the sample size. The experiments were not randomized. Three tumors with low tumor purity were removed from genomic analyses due to high contamination by non-tumor cells that do not allow us to assess mutational and gene expression profiles. For spatial transcriptomics, low-quality spots were filtered out based on a very low number of genes detected in patient #3115 indicating a detachment of the tissue in a small area. For methylation analyses in MS-MLPA, we excluded one probe from the analysis targeting IC1 (H19.11.001.976583) because its distribution did not discriminate between samples with and without gain of methylation IC1. In snRNAseq, cells with more than 5% mitochondrial genes were removed. We kept only cells with a minimum of 1000 genes detected in each cell. We removed a cluster of cells with high mitochondrial genes, high ambient RNA, and low features in each patient. Detailed statistical analyses are specified in each figure legend.

### Reporting summary

Further information on research design is available in the Nature Portfolio Reporting Summary linked to this article.

### Supplementary information

Supplementary Information

Description of Additional Supplementary Files

Supplementary Data 1

Supplementary Data 2

Supplementary Data 3

Supplementary Data 4

Supplementary Data 5

Supplementary Data 6

Supplementary Data 7

Supplementary Data 8

Supplementary Data 9

Reporting Summary

### Source data

Source Data

## Data Availability

The raw data from this study, including WGS, WES, RNAseq, RRBS, snRNAseq, and visium datasets have been archived in the EGA database under accession codes EGAS00001005108, EGAS00001003837, and EGAS00001006692. Raw sequence data (FASTQ files) access is available upon successful application to the ‘Data Access Committee’ EGAC00001002924 according to the EGA general guidance. Access to these datasets is restricted to comply with European data protection regulations and to protect the privacy and rights of individuals whose data may be included in these datasets. When the application is successful, access is granted for the duration of the approval project. Any utilization of the data for a different project will require prior approval through a new agreement. A response to all initial requests should be done in 6 weeks. The processed spatial transcriptomics and snRNAseq data can be found in the Figshare repository (10.6084/m9.figshare.23552595), and source data are provided along with this paper. The fetal liver methylation publicly available data used in this study are available in the GEO database under accession code GSE61278^21^. The remaining data are available within the Article, Supplementary Information, or Source Data file. Source data are provided in this paper.

## Code availability

All custom scripts generated for this study are available on Github: https://github.com/FunGeST/Preneoplastic_liver_colonization.
