# Supplementary material for: Preneoplastic liver colonization by 11p15.5 altered mosaic cells in young children with hepatoblastoma
Source: Nat Commun. 2023 Nov 6;14:7122. doi: 10.1038/s41467-023-42418-9 (PMC10628292; doi:10.1038/s41467-023-42418-9)
Supplement: Supplementary file 1 — Supplementary Information [file 41467_2023_42418_MOESM1_ESM.pdf]

Supplementary Figure 1

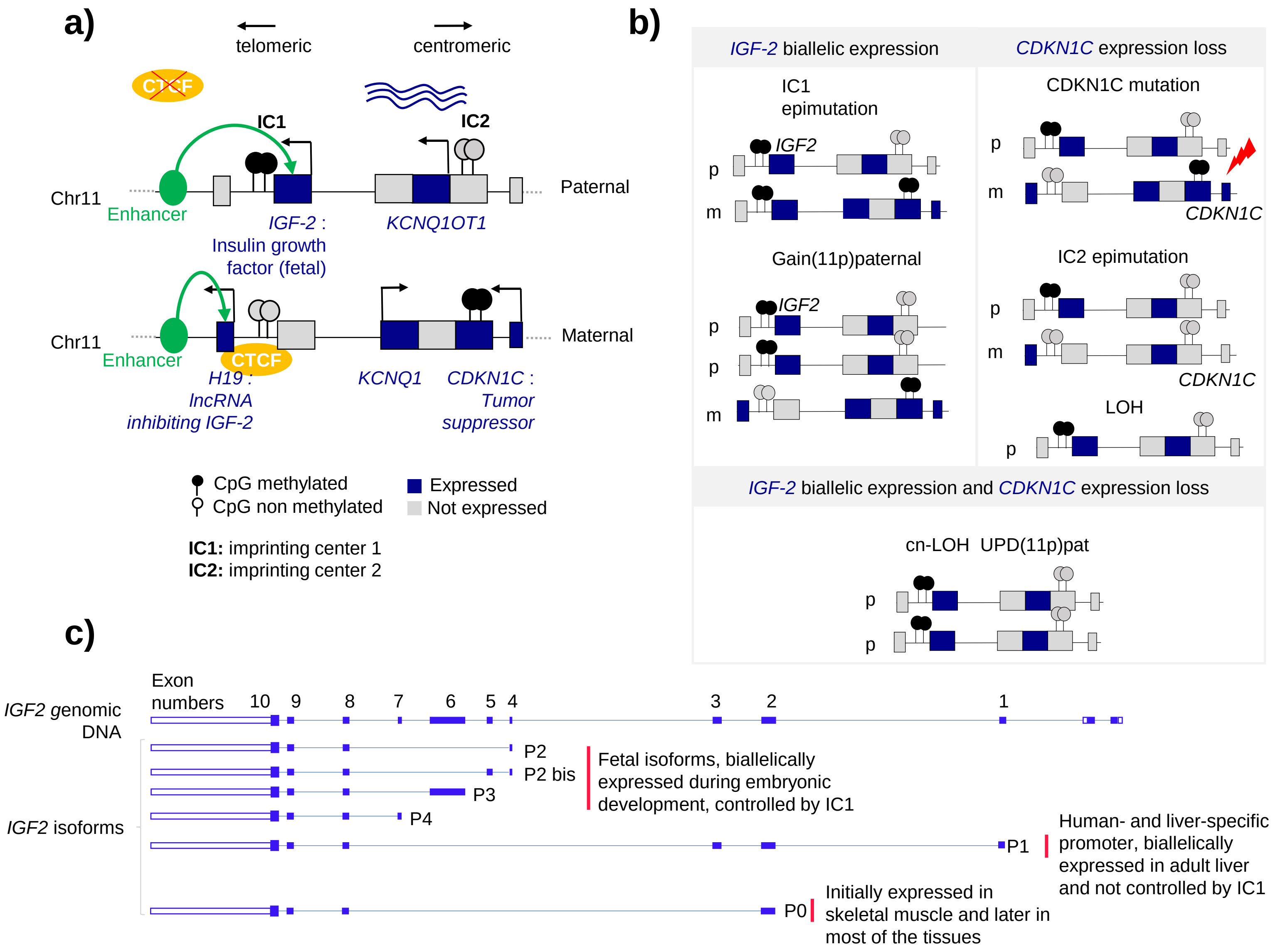

Supplementary figure 1. 11p15.5 locus organization, alterations in BWS syndrome and *IGF2* promoter usage. a) Scheme representing 11p15.5 locus organization where Imprinting center 1 (IC1) and imprinting center 2 (IC2) are differentially methylated on the maternal and paternal alleles. Consequently, *IGF2* and *KCNQ1OT1* are expressed on the paternal allele whereas *H19*, *KCNQ1* and *CDKN1C* are expressed on the maternal allele. Given that *IGF2* and *H19* share the same enhancers, when IC1 is unmethylated on the maternal allele, *H19* promoter binds its enhancers through a loop mediated by CCCTC binding factor (*CTCF*) which leads to *H19* expression. However, on the paternal allele, IC1 is methylated which prevents the binding of CTCF and *IGF2* is expressed through the binding to its distal enhancers. b) Schematic representation of the different mechanisms of 11p15.5 locus alterations in BWS syndrome. IC1 epimutation and a paternal duplication lead to the expression of two copies of *IGF2*. A mutation occurring in *CDKN1C* gene, an IC2 epimutation (loss of methylation) and a loss of heterozygosity (LOH) can alter *CDKN1C* expression. Finally, a copy-neutral LOH (cn-LOH) lead both to the expression of two copies of *IGF2* and a putative change in *CDKN1C* expression. c) Schematic representation of *IGF2* genomic region, isoforms and promoters (P0-P4). Exon numbers are indicated on genomic DNA. UPD(11p)pat: Uniparental disomy of paternal 11p allele, LOH: loss of heterozygosity, IC1/IC2: Imprinting center 1/2, P0-P4: Promoter 0-4.

Supplementary Figure 2

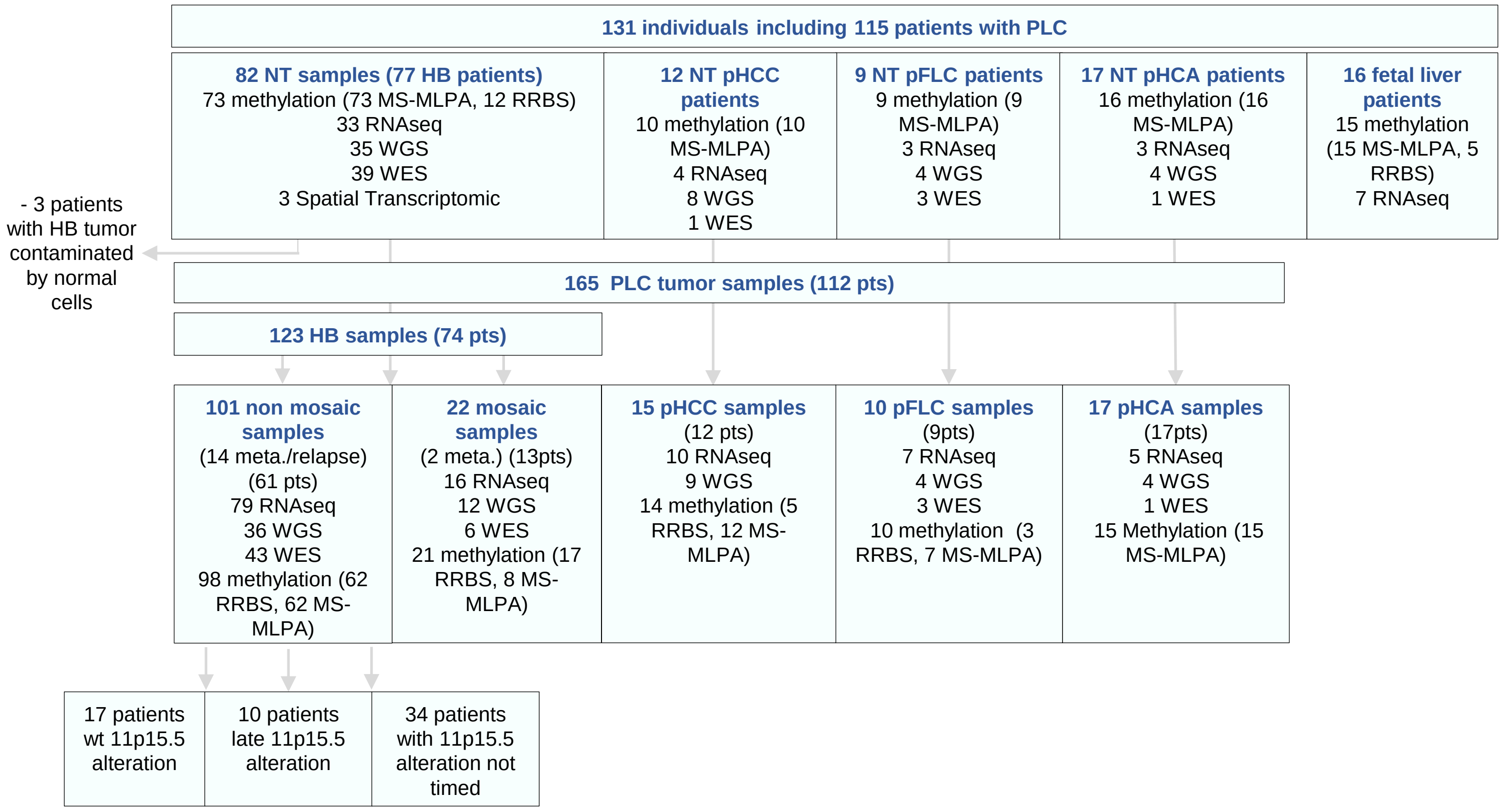

Supplementary Figure 2. Detailed description of pediatric liver non tumor and tumor cohorts. The number of patients and samples is indicated for each technique. pts : patients, WGS : Whole-genome sequencing, WES : Whole-Exome sequencing, MS-MLPA : Methylation-specific multiplex ligation dependent probe amplification, RNAseq : RNA sequencing, NT : non tumor, HB : hepatoblastoma, pHCC : pediatric hepatocellular carcinoma, pFLC : pediatric fibrolamellar carcinoma, pHCA : pediatric hepatocellular adenoma, PLC : pediatric liver cancer, meta. : metastasis, pts: patients, wt: wild-type.

Supplementary Figure 3

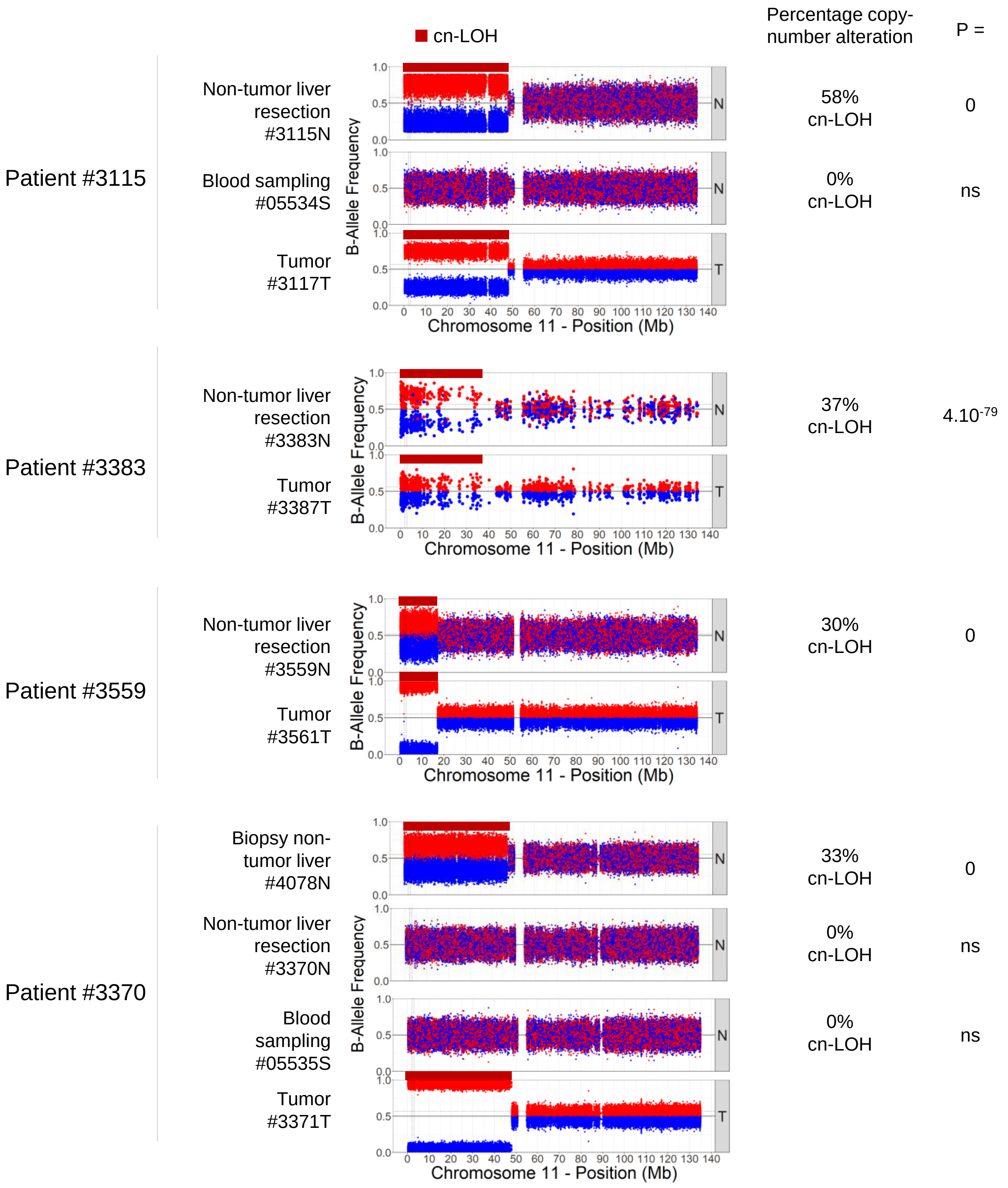

Supplementary figure 3. B-allele frequency at chromosome 11 of heterozygous SNPs in 4 mosaic patients. SNPs with a BAF higher than 0.5 in the tumor are coloured in red whereas SNPs with a BAF lower than 0.5 in the tumor are blue. The amplitude of 11p15.5 locus cn-LOH indicates the proportion of cells altered. Binomial tests were performed.

Supplementary Figure 4

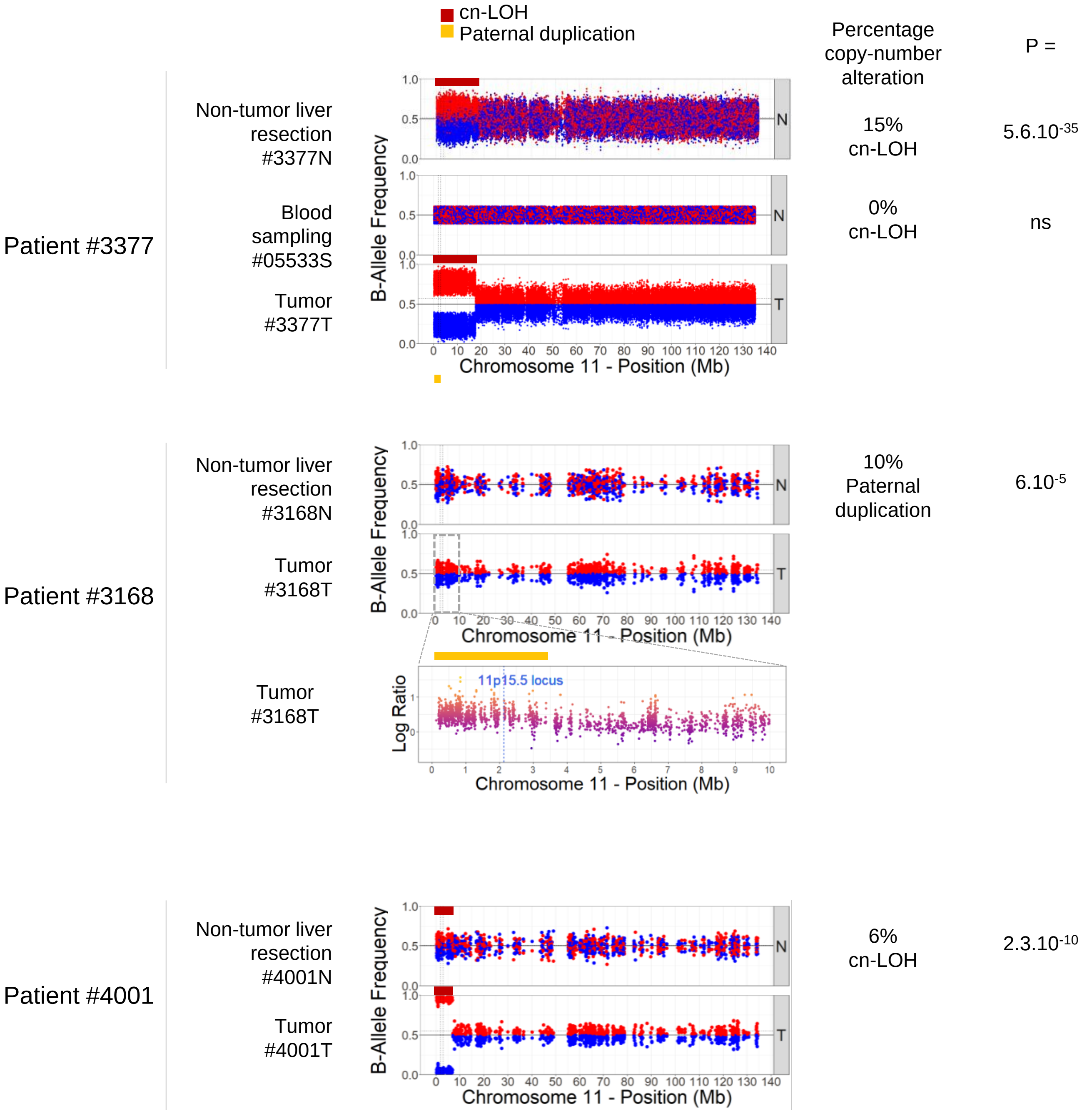

Supplementary figure 4. B-allele frequency at chromosome 11 of heterozygous SNPs in 3 mosaic patients. SNPs with a BAF higher than 0.5 in the tumor are coloured in red whereas SNPs with a BAF lower than 0.5 in the tumor are blue. The amplitude of 11p15.5 locus cn-LOH indicates the proportion of cells altered. Binomial tests were performed.

Supplementary Figure 5

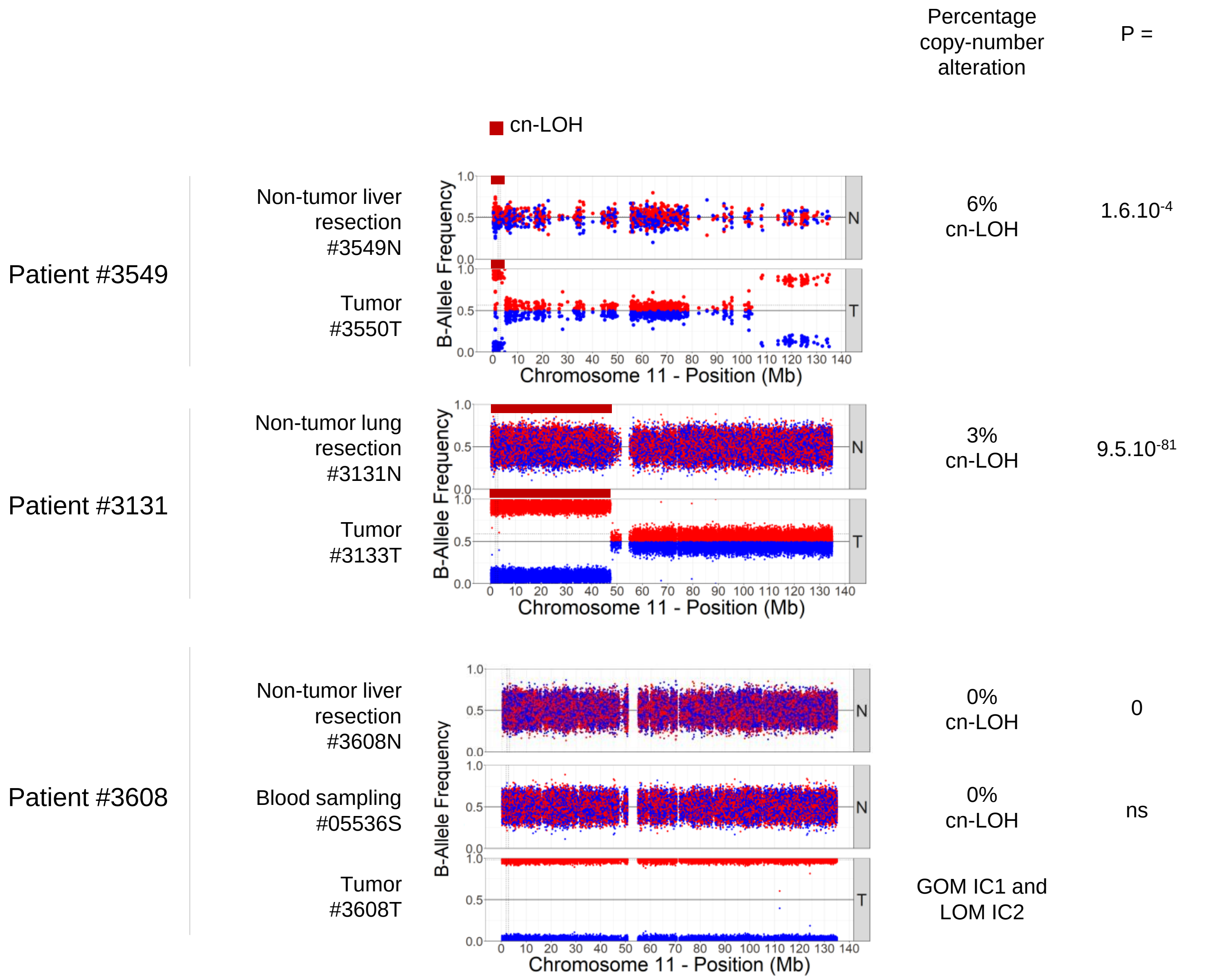

Supplementary figure 5. B-allele frequency at chromosome 11 of heterozygous SNPs in 3 mosaic patients. SNPs with a BAF higher than 0.5 in the tumor are coloured in red whereas SNPs with a BAF lower than 0.5 in the tumor are blue. The amplitude of 11p15.5 locus cn-LOH indicates the proportion of cells altered. Binomial tests were performed.

Supplementary Figure 6

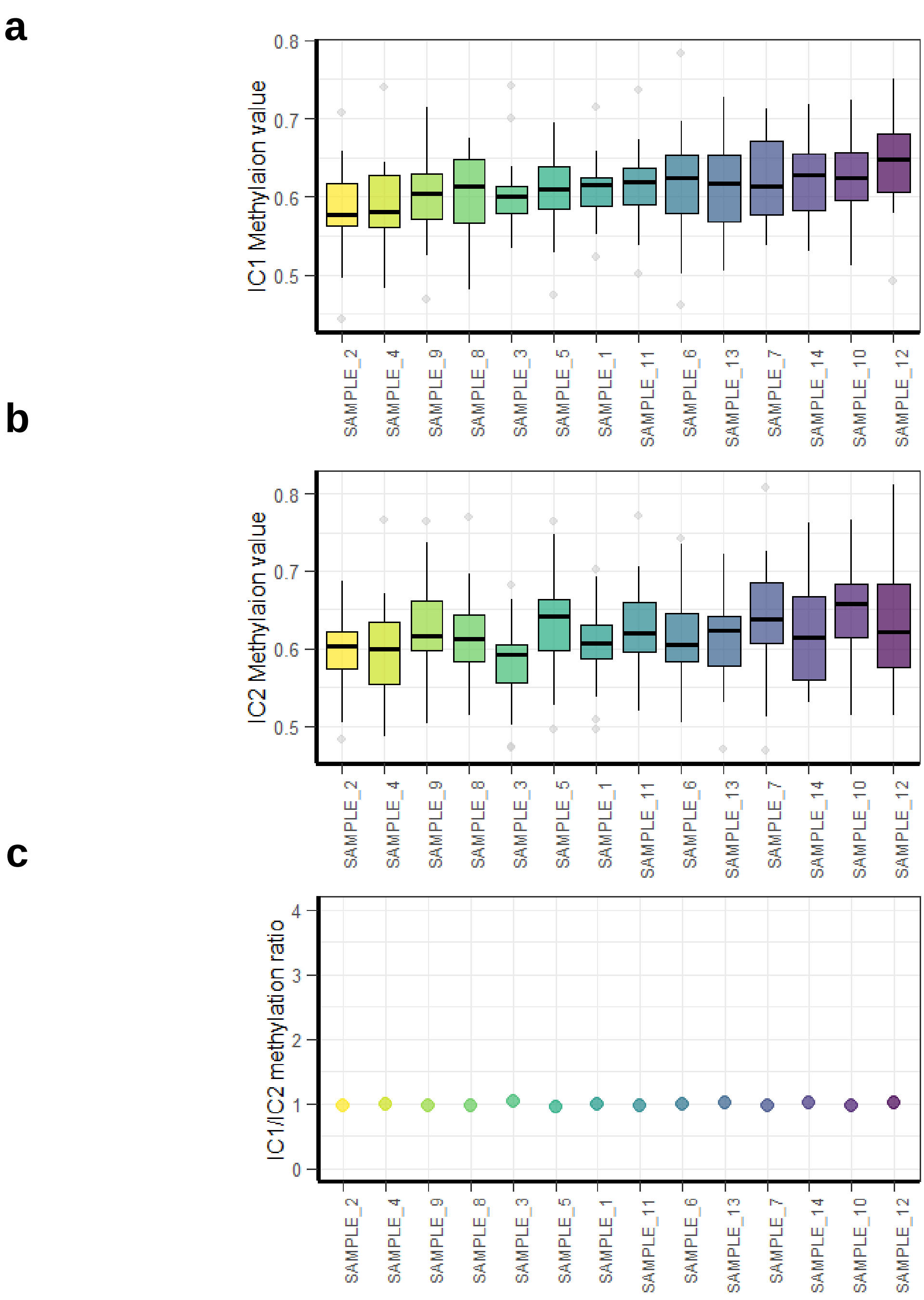

Supplementary figure 6. IC1 and IC2 methylation levels in 14 fetal livers from Bonder *et al.* BMC Genomics 2014*.* a) Methylation levels in 22 CpG located inside IC1 and b) 26 CpG inside IC2 region. c) IC1/IC2 methylation ratio indicates an absence of an 11p15.5 locus alteration in 14 fetal liver samples published in Bonder *et al.* BMC Genomics 2014[1]. Source data are provided as a Source Data file.

Supplementary Figure 7

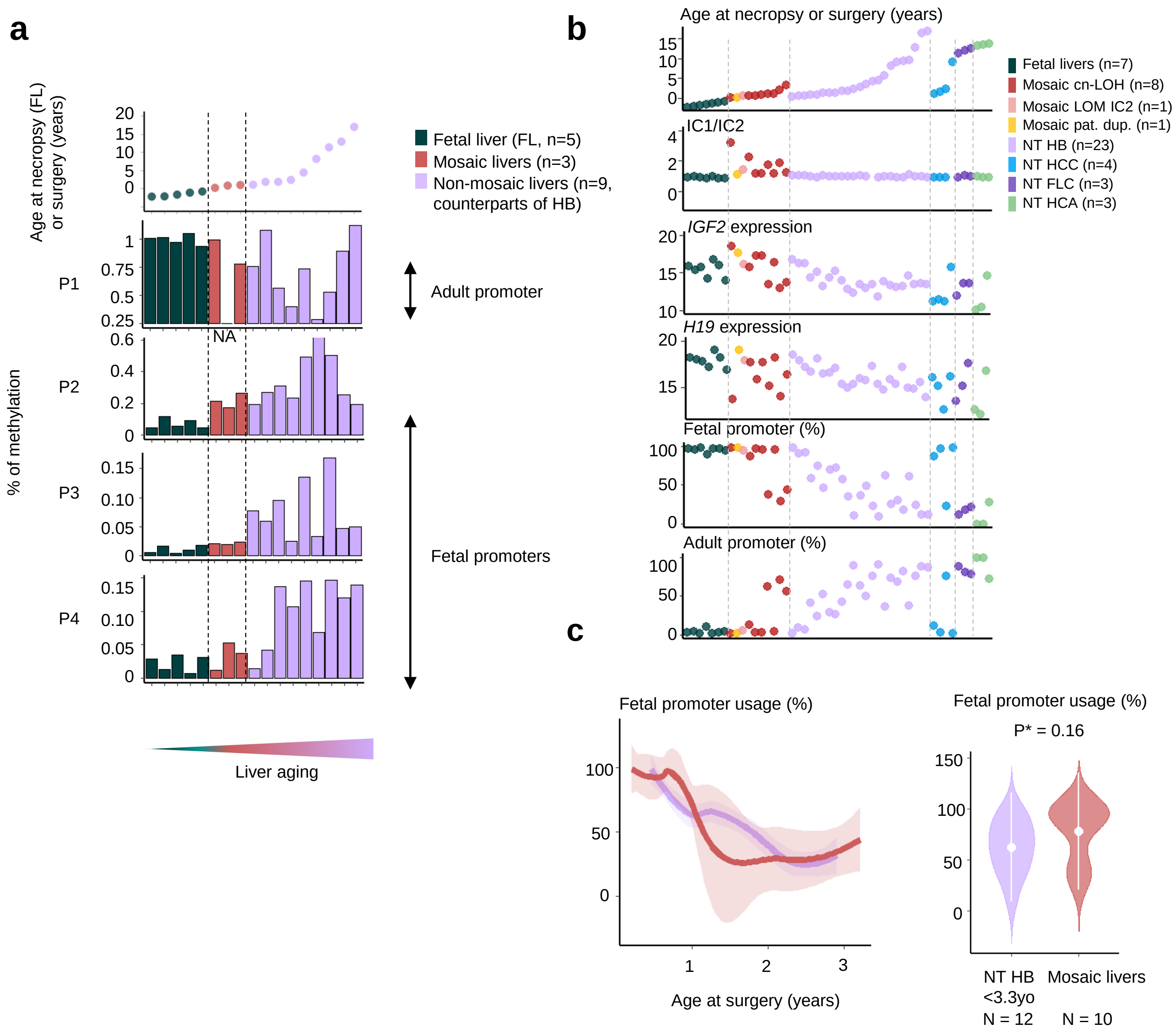

Supplementary figure 7. *IGF2* promoter usage in mosaic and non-mosaic livers

a) *IGF2* promoters (P1 : adult, P2-P3-P4: fetal) methylation status in 5 fetal livers, 3 mosaic and 9 non-mosaic post-natal livers. Methylation values were obtained from RRBS sequencing. *IGF2* fetal promoters are progressively methylated with increasing age at surgery consistent with a progressive switch from fetal to adult promoter usage. b) Consequences of 11p15.5 locus mosaic alterations on IC1/IC2 methylation levels, *IGF2* and *H19* expression levels and *IGF2* promoter usage in 50 non-tumor liver samples. c) Evolution of fetal promoter usage with age at surgery in 10 mosaic and 12 non-mosaic livers younger than 3.3 years old. Loess regression and two-sided Wilcoxon test were performed. FL: fetal liver, cn-LOH: copy-neutral LOH, LOM IC2: loss of methylation IC2, yo: years old, NT: non-tumor counterpart. Source data are provided as a Source Data file.

Supplementary Figure 8

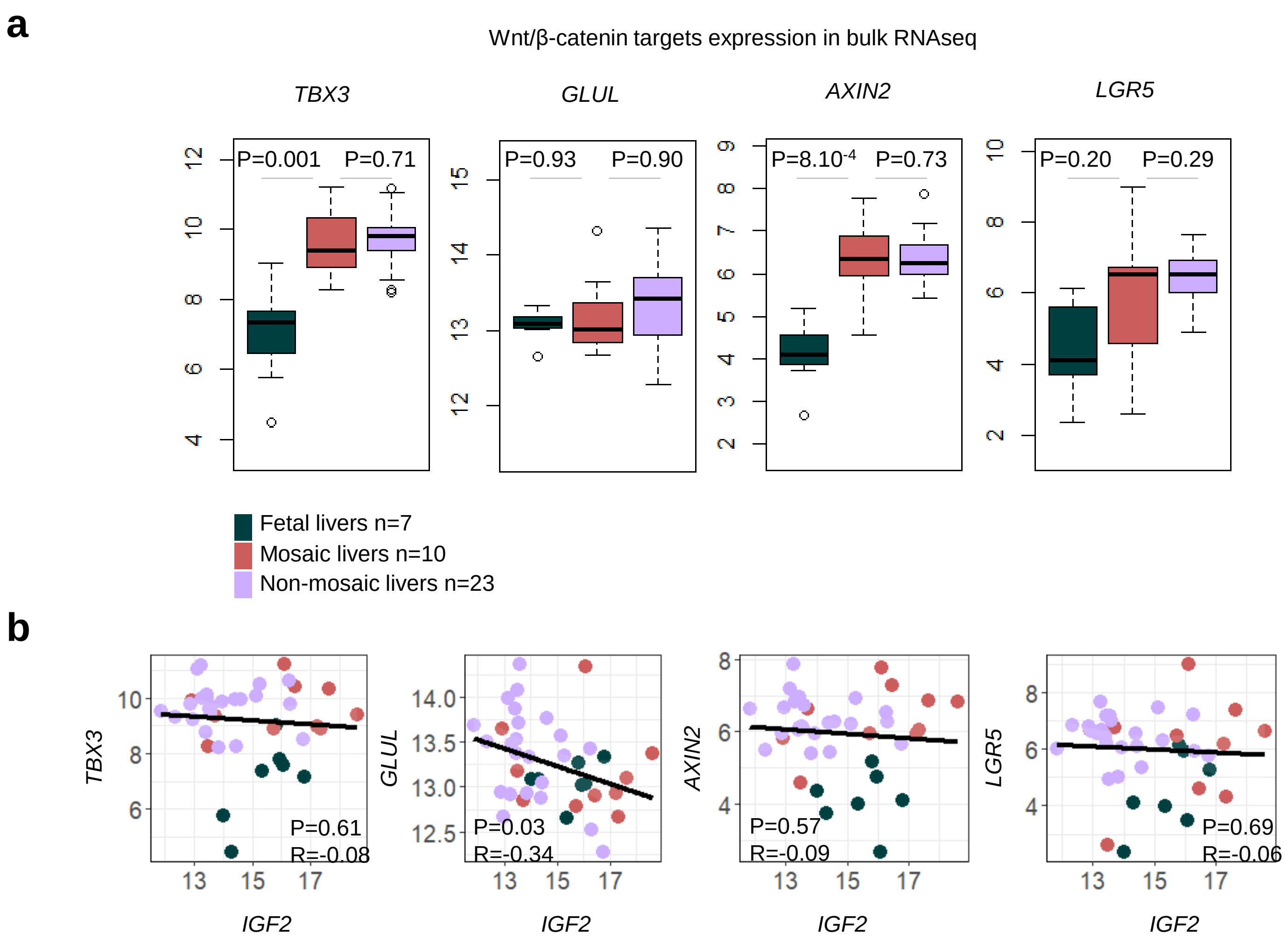

Supplementary figure 8. Lack of Wnt/β-catenin pathway activation in mosaic non-tumor livers.
a) Bulk RNAseq expression of 4 markers (*TBX3*, *GLUL*, *AXIN2* and *LGR5*) of Wnt/β-catenin pathway activation in 7 fetal livers, 10 mosaic and 23 non-mosaic livers. Statistical tests: Limma differential expression analysis was performed using two-sided moderated t-test adjusted for age at surgery. b) Correlation between *IGF2* and β-catenin target genes expression levels (variance stabilized transformation) in 7 fetal livers, 10 mosaic and 23 non-mosaic livers. Pearson correlation test was performed. Source data are provided as a Source Data file.

Supplementary Figure 9

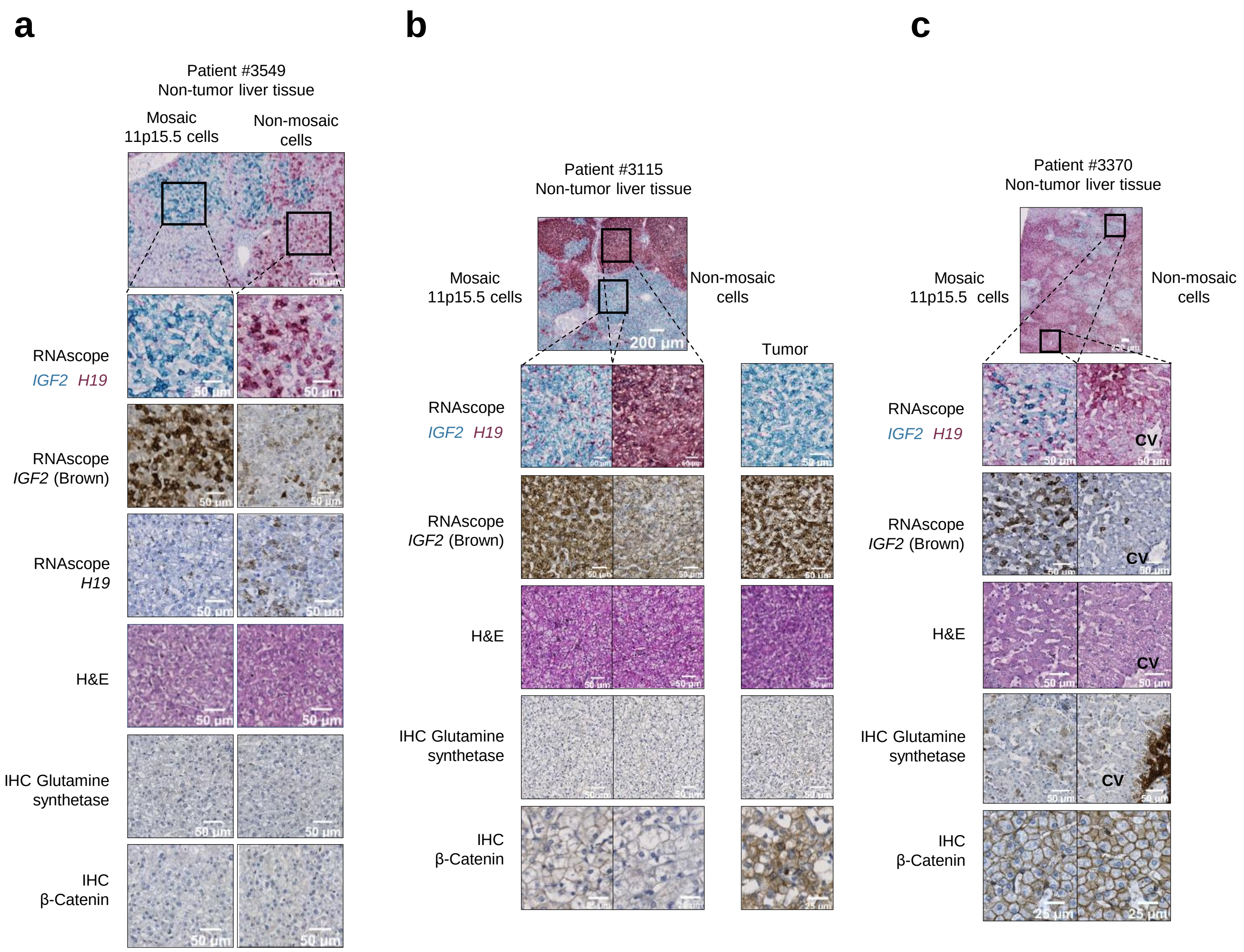

Supplementary figure 9. *In situ* visualization of cn-LOH alteration in mosaic patients #3549 (a), #3115 (b) and #3370 (c). Singleplex and duplex RNAscope stainings of *IGF2* and *H19* were performed as well as Hematoxylin and eosin, Glutamine synthetase and β-catenin immunostainings. Mosaic patches with 11p15.5 cn-LOH displayed *IGF2* overexpression (blue) and *H19* expression loss (red) in duplex RNAscope assay with no effect on Glutamine synthetase and β-Catenin expression. Non-mosaic hepatocytes expressed both *IGF2* and *H19* known to originate from paternal and maternal alleles, respectively. Scale bars represented in a) 200µM and 50µM, b) 25µM, 50µM and 200µM, c) 25µM, 50µM and 200µM.

Supplementary Figure 10

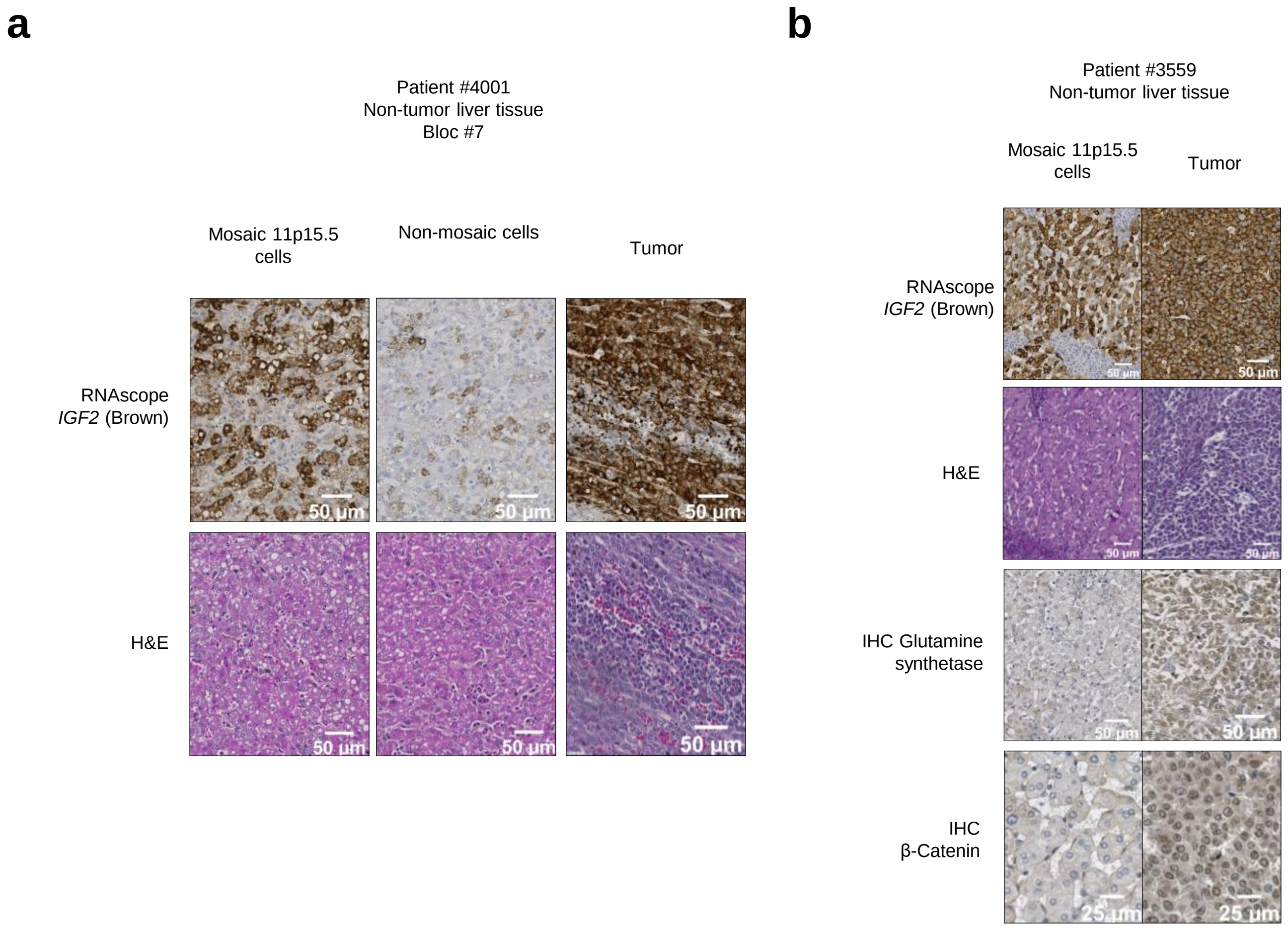

Supplementary figure 10. *In situ* visualization of cn-LOH alteration in two FFPE slides (#7 and #15) from mosaic patients #4001 and one slide from patient #3559. Singleplex RNAscope stainings of *IGF2* were performed as well as Hematoxylin and eosin, Glutamine synthetase and β-catenin immunostainings. Mosaic patches with 11p15.5 cn-LOH displayed *IGF2* overexpression (brown) in RNAscope singleplex assay with no effect on Glutamine synthetase and β-Catenin expression. Scale bars represented in a) 50µM and b) 25µM and 50µM.

Supplementary Figure 11

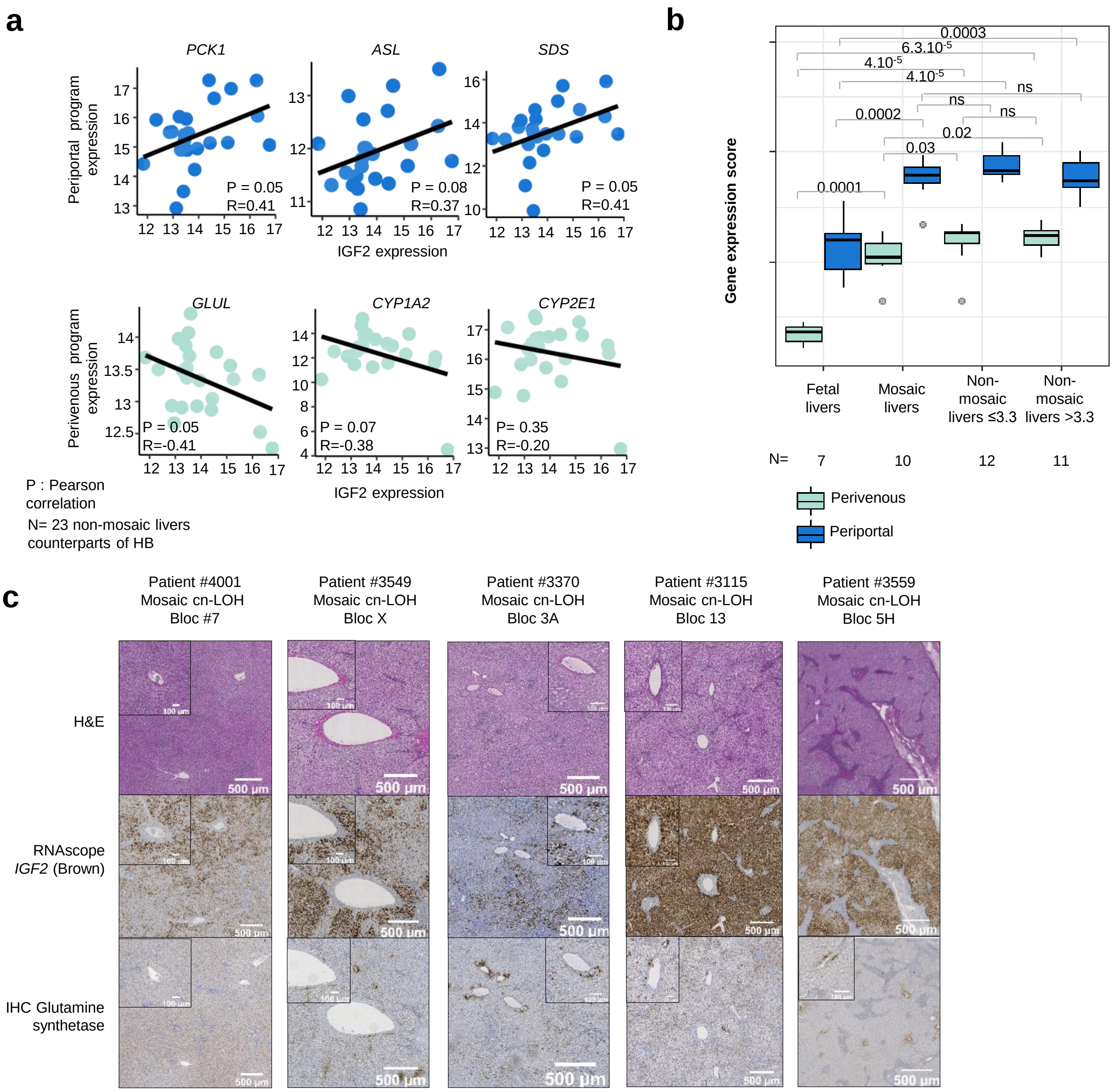

Supplementary figure 11. Zonation disturbances in mosaic 11p15.5 livers. a) Correlation between *IGF2* expression levels and periportal/perivenous zonation markers in bulk RNAseq in 23 non-mosaic livers. Pearson correlations were performed. b) Perivenous and periportal gene expression scores in fetal livers, mosaic and non-mosaic livers. Scores were calculated based on the mean expression of periportal (*HAL*, *SDS*, *PCK1*, *ASL*, *ASS1*, *CPS1*) and perivenous genes (*GLUL*, *LGR5*, *AXIN2*, *CYP2E1*, *CYP1A2*, *OAT*). Two-sided Wilcoxon statistical tests were performed. c) Representative images of GS attenuation around centrilobular veins in mosaic liver FFPE sections. Scale bars, 100µM and 500µM. Source data are provided as a Source Data file.

Supplementary Figure 12

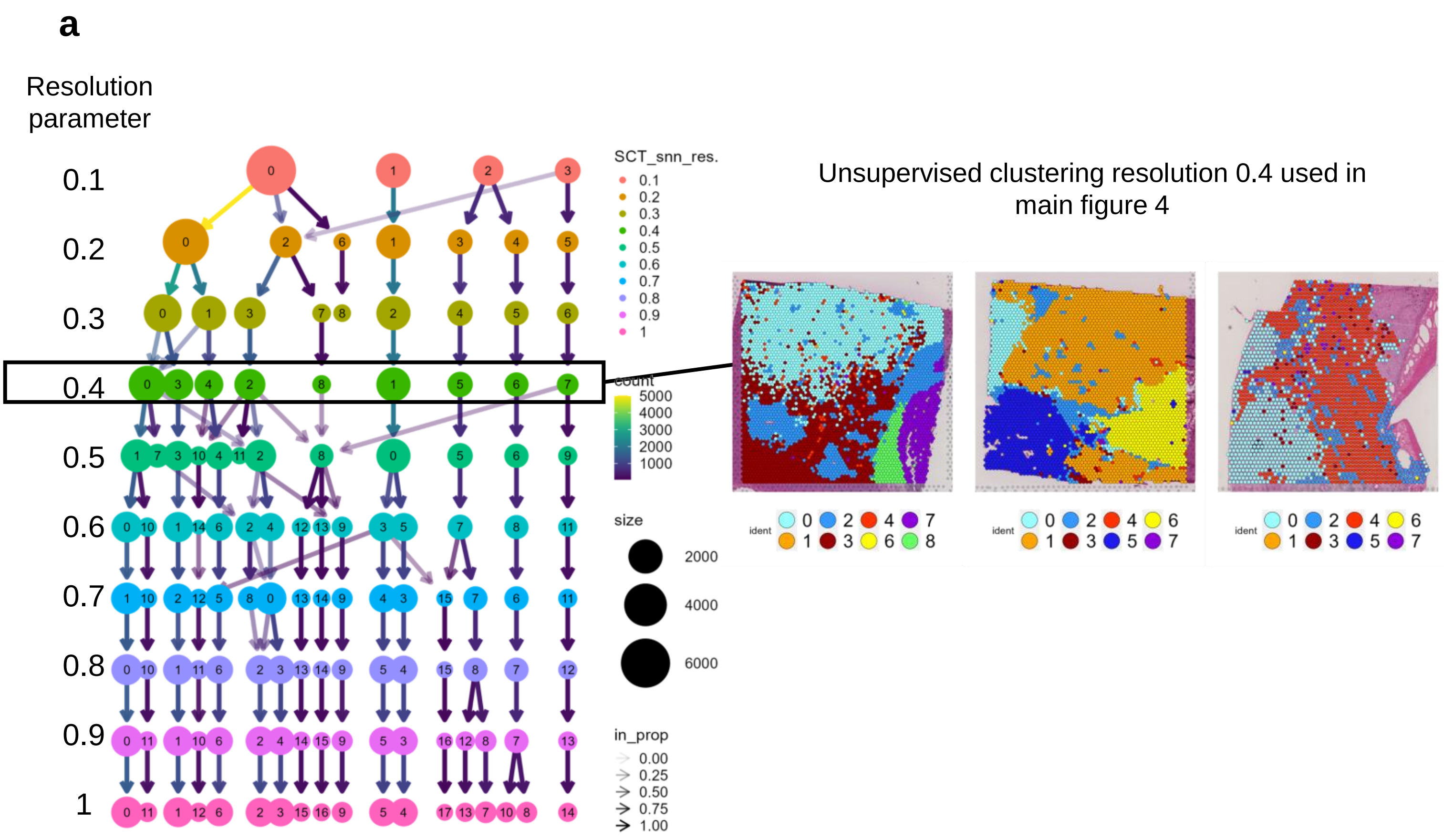

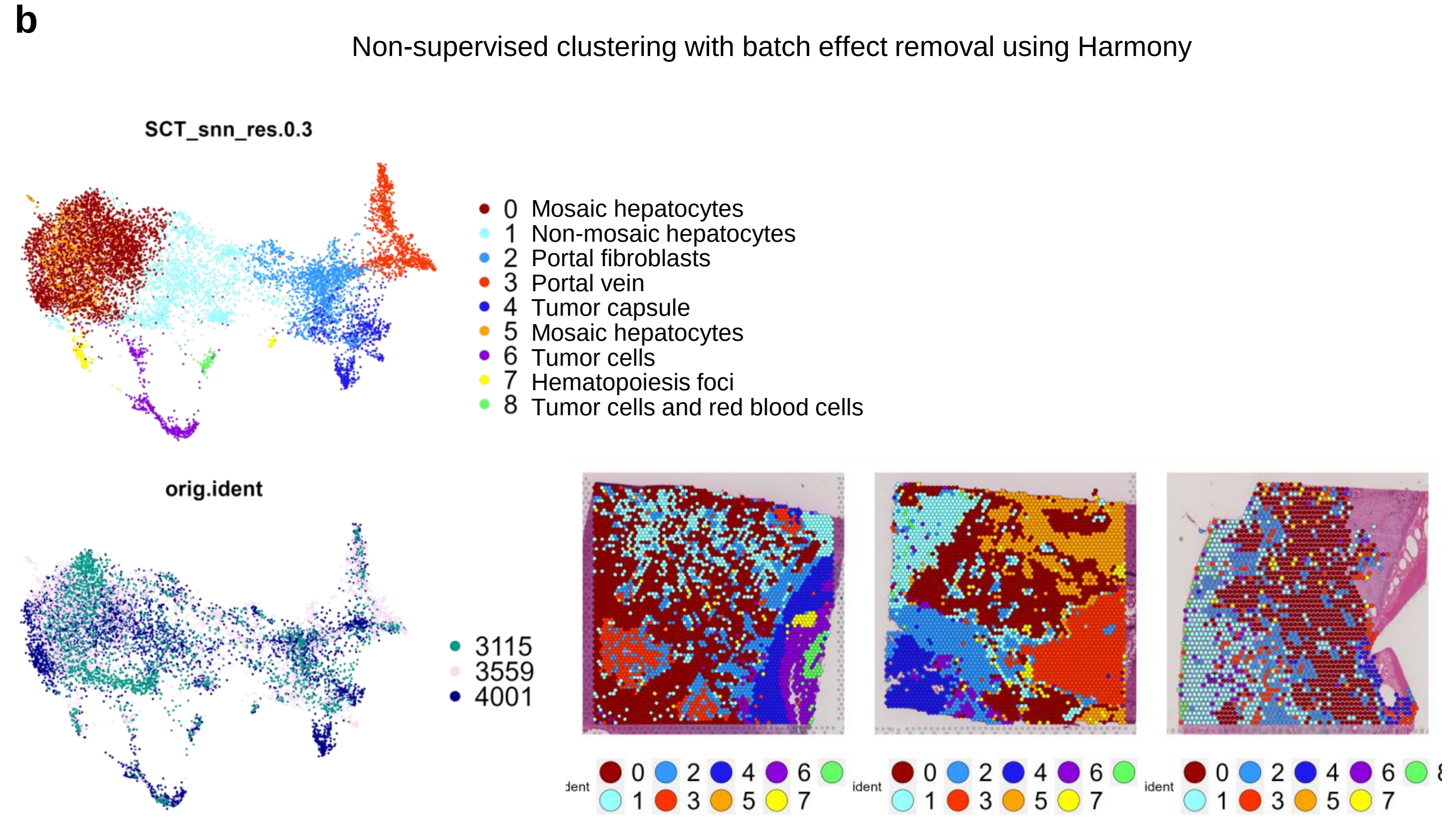

Supplementary figure 12. Non-supervised clustering analysis of 3 spatially resolved FFPE liver slides from 3 patients with 11p15.5 locus mosaicism. a) Non-supervised clustering analysis on the merged object (Patients #4001, #3559 and #3115 analysed together) displayed main Figure 4 identified 9 robust clusters. On the left, the clustering tree with *clustree* function shows robustness of the clusters identified. The size of each node reflects the number of visium spots and their color correspond to the resolution used. Edges color reflects the number of visium spots. b) Non-supervised clustering of the merged *seurat* object after batch effect removal using *Harmony* R package. Similar clusters were obtained with and without batch effect removal.

Supplementary Figure 13

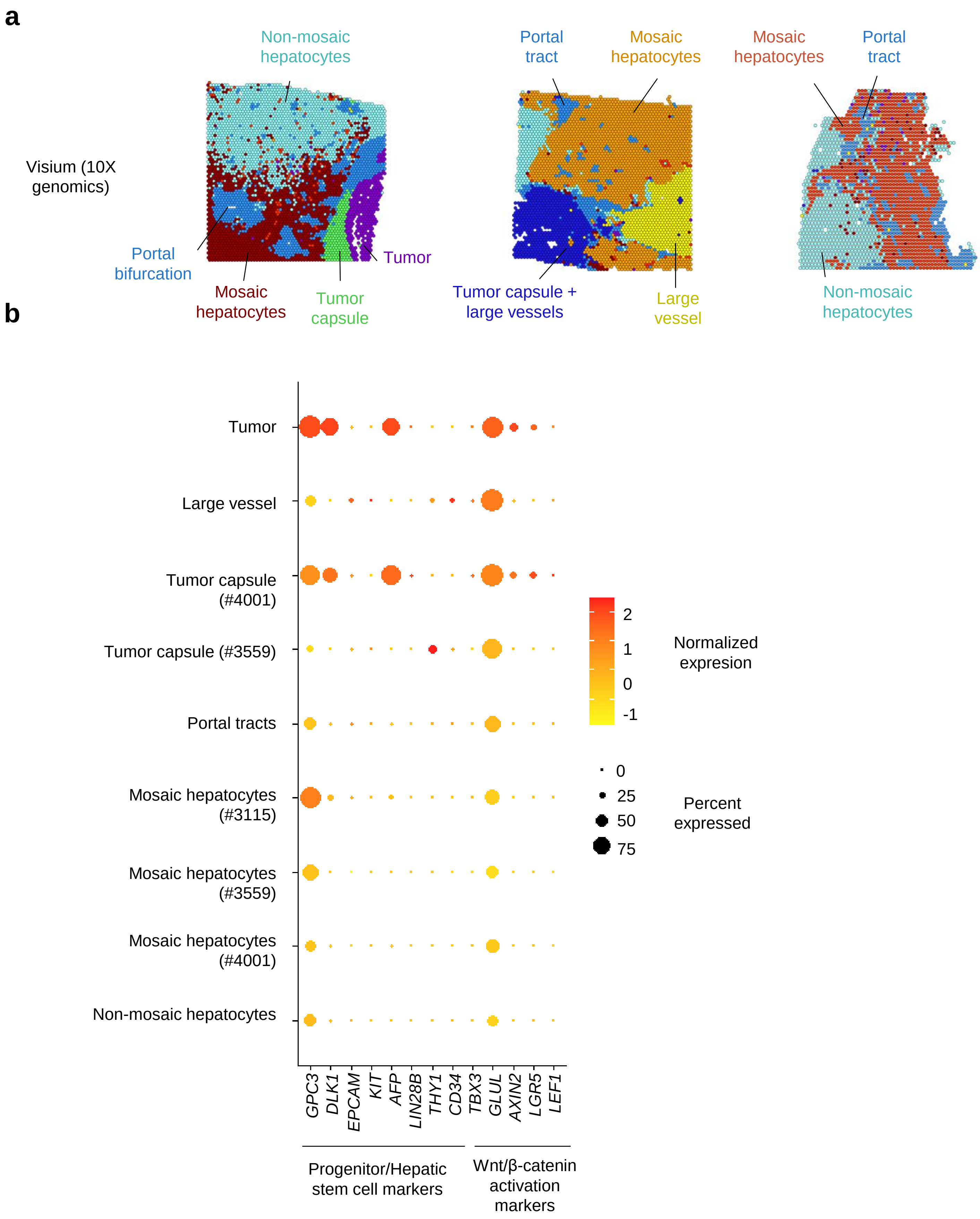

Supplementary figure 13. Expression of progenitor/stem cell markers in 3 spatially resolved mosaic livers. a) Unsupervised clustering of 3 non-tumor livers from patients #4001, #3559 and #3115. b) Expression of progenitor/stem cell markers and Wnt/β-catenin activation markers in clusters from spatial transcriptomics analysis. Source data are provided as a Source Data file.

Supplementary Figure 14

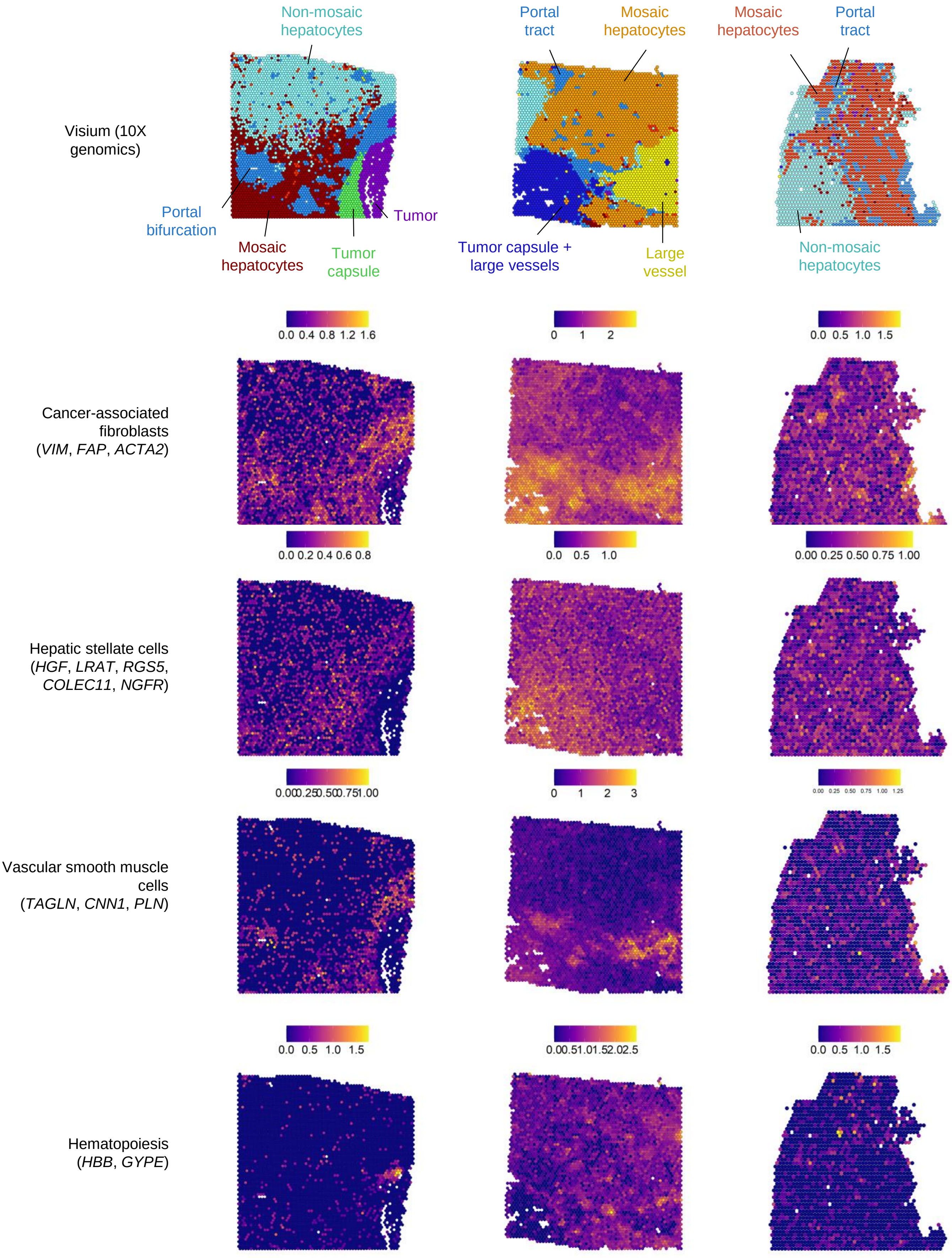

Supplementary figure 14. Spatial genes expression signatures in non-tumor liver from 3 patients with 11p15.5 mosaic cn-LOH. Mean gene expression was calculated for each gene set in order to derive a score. Genes involved in each gene set are indicated in brackets. Source data are provided as a Source Data file.

Supplementary Figure 15

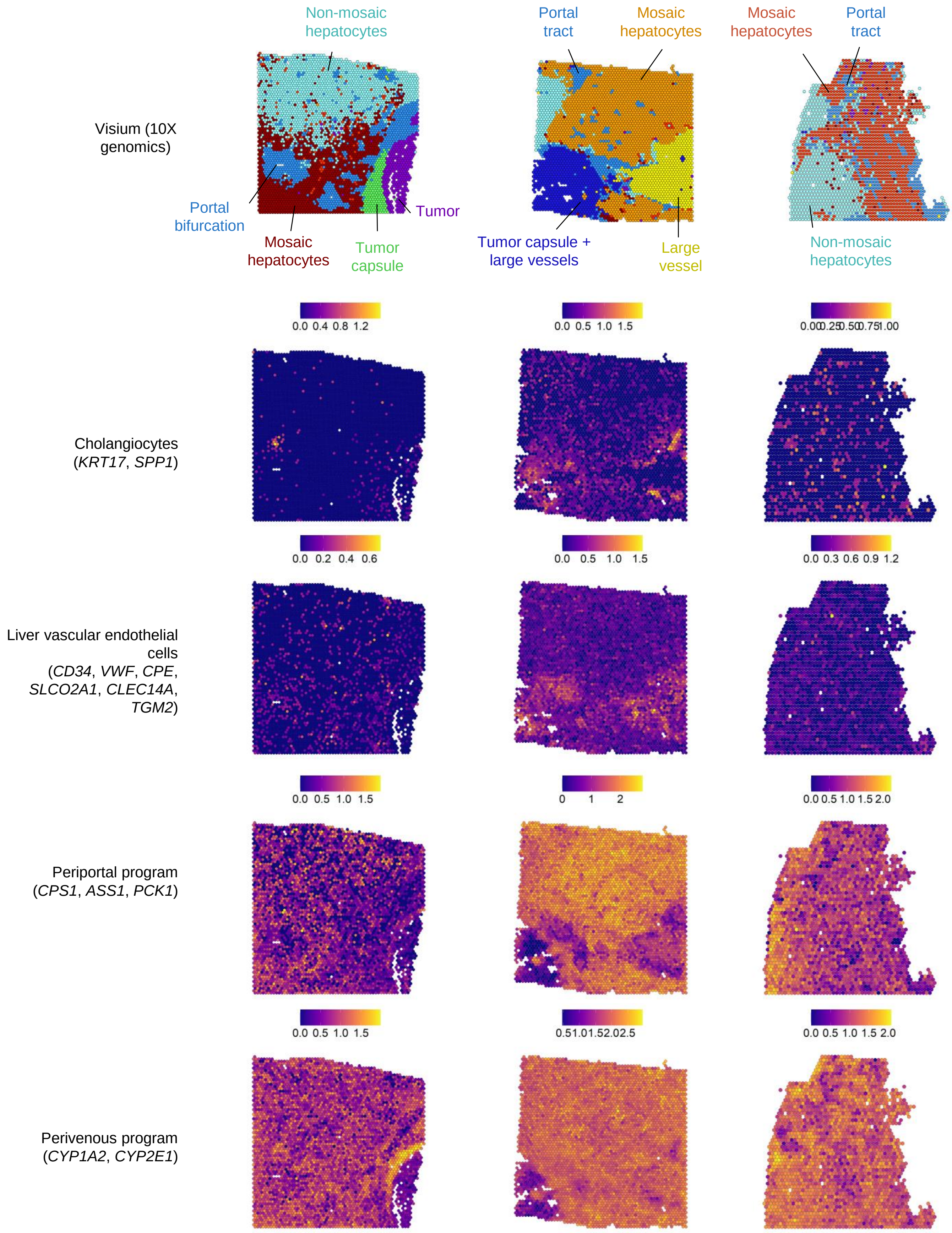

Supplementary figure 15. Spatial genes expression signatures in non-tumor liver from 3 patients with 11p15.5 mosaic cn-LOH. Mean gene expression was calculated for each gene set in order to derive a score. Genes involved in each gene set are indicated in brackets. Source data are provided as a Source Data file.

Supplementary Figure 16

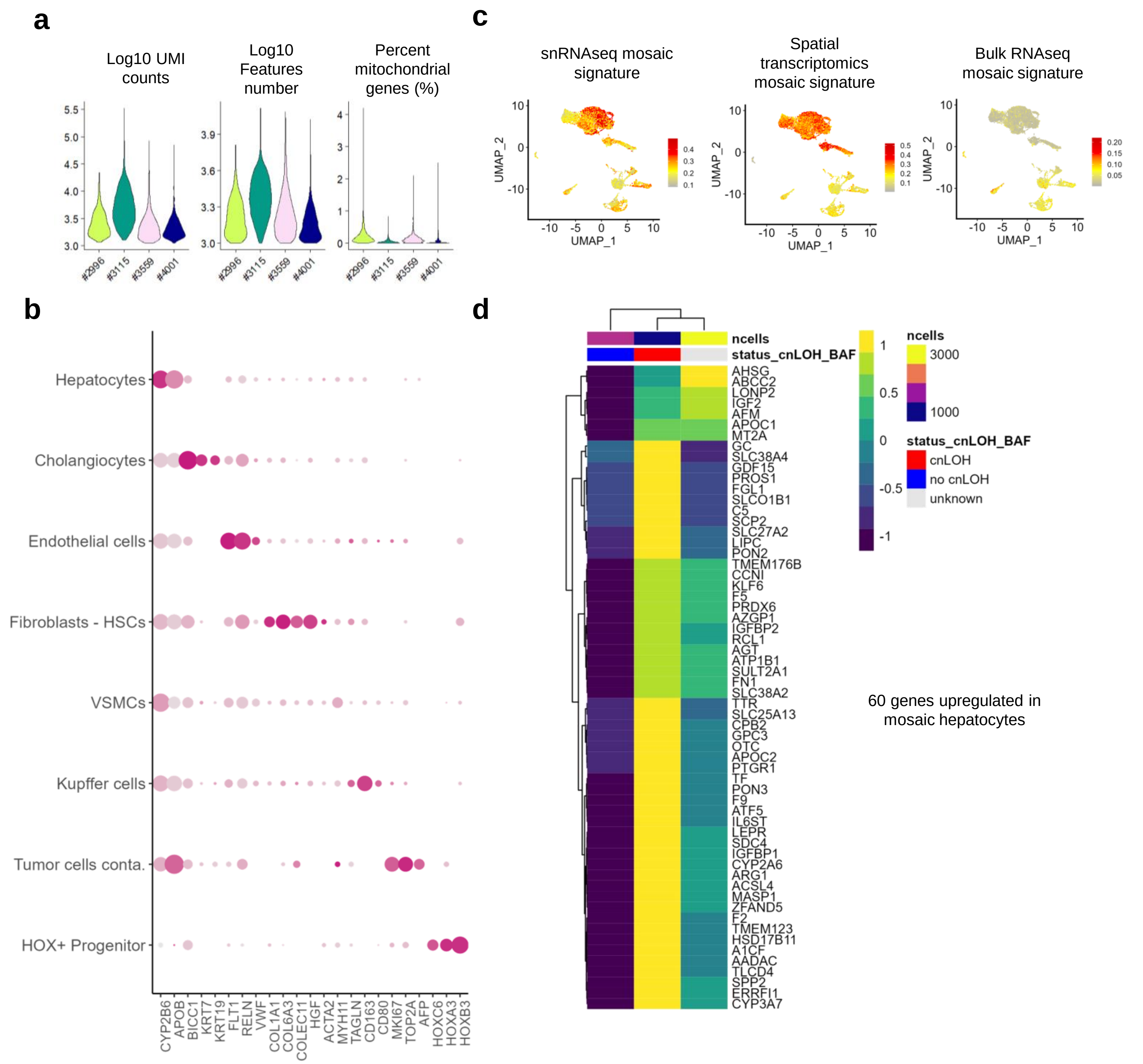

Supplementary figure 16. Visualization of marker genes expression identified in single-nucleus RNAseq from 4 patients (#3115, #4001, #3559 and #2996). a) Number of UMI counts, genes detected per cell and percentage of mitochondrial genes in each sample after performing quality controls. b) Gene expression of typical markers of cell populations from the 4 patients. c) Visualization of mosaic transcriptomic signatures derived from snRNAseq, spatial transcriptomics and bulk RNAseq. d) Heatmap representation of 60 commonly upregulated genes in mosaic hepatocytes in snRNaseq and spatial transcriptomics.

Supplementary Figure 17

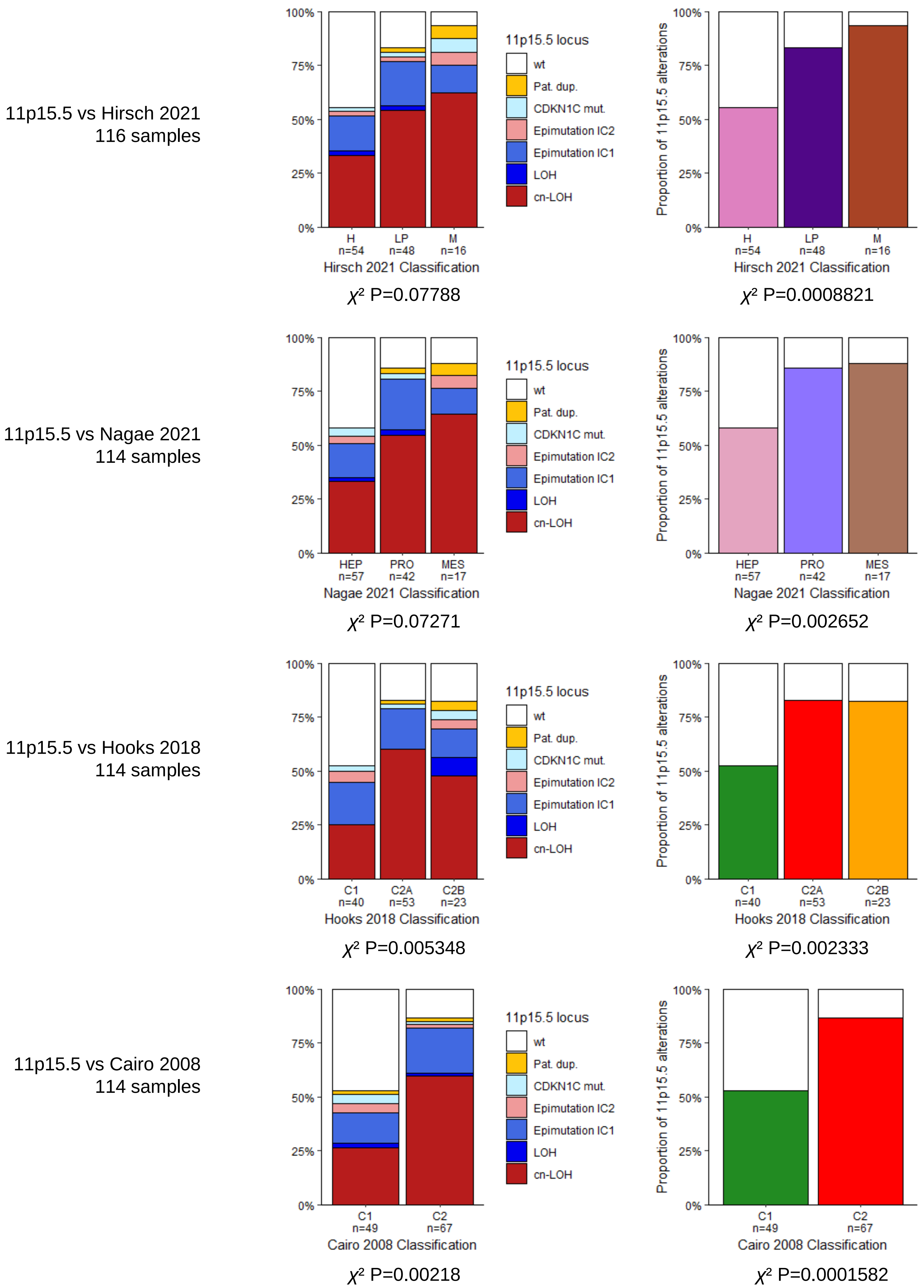

Supplementary figure 17. HB tumors transcriptomic classification and association with 11p15.5 locus alteration. Proportion of HB tumors in published classifications from Cairo *et al.*, 2008[2] (C1/C2, 114 tumor samples), Hooks *et al.* 2018[3] (C1/C2A/C2B, 114 tumor samples), Nagae *et al.*, 2021[4] (Hep/Pro/Mes, 114 tumor samples) and Hirsch *et al.* 2021[5] (H, LP, M, 116 tumor samples). Two-sided χ^2 statistical test was performed. Source data are provided as a Source Data file.

Supplementary Figure 18

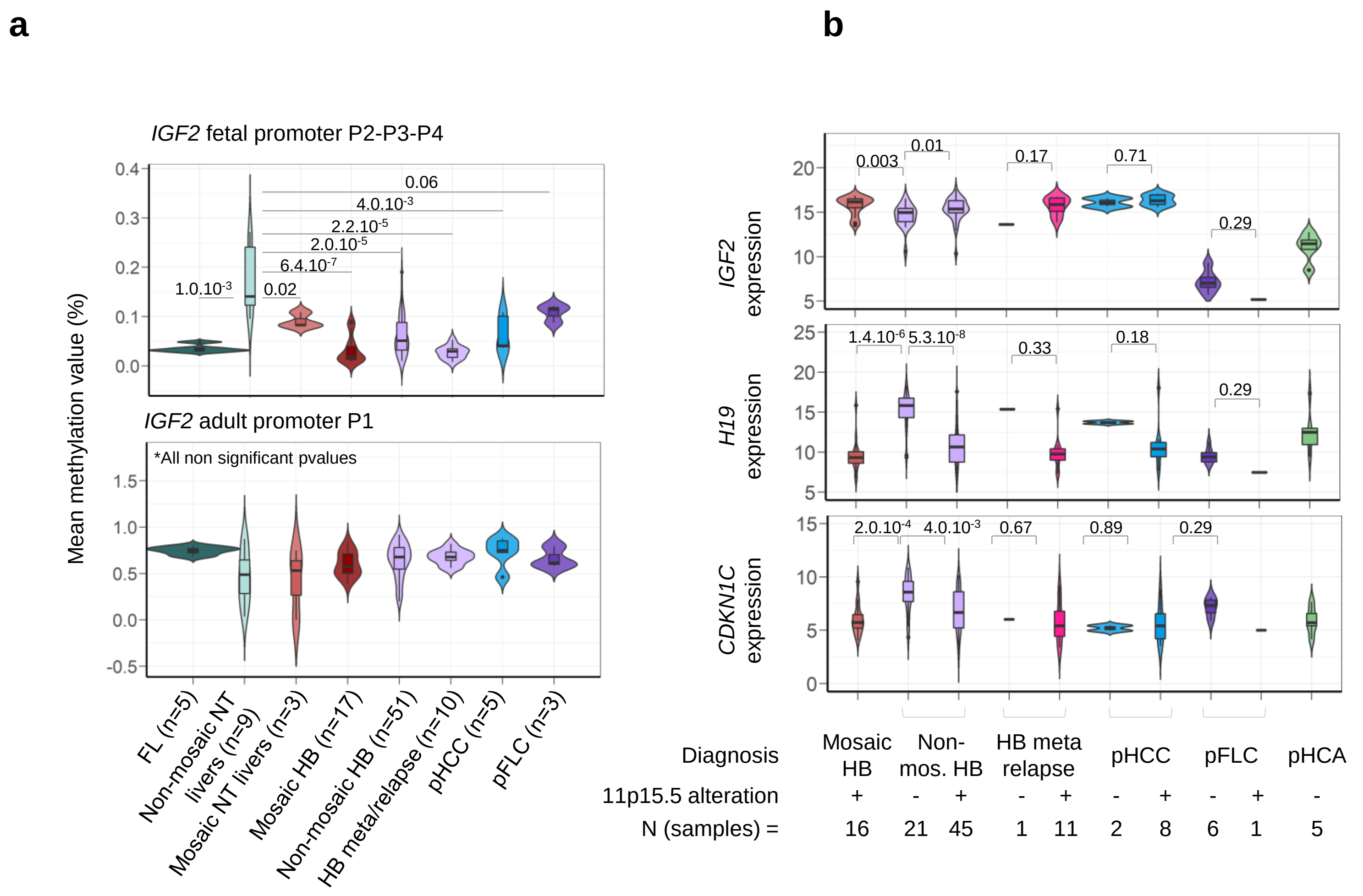

Supplementary figure 18. Regulation of *IGF2*, *H19*, *CDKN1C* imprinted genes expression. a) *IGF2* promoter methylation in 5 fetal livers, 12 non-tumor livers and 86 pediatric liver tumors. A mean of fetal liver promoters (P2, P3, P4) methylation levels was performed. b) RNAseq bulk gene expression of *IGF2*, *H19* and *CDKN1C* in 116 pediatric liver tumors. Two-sided Mann-Whitney-Wilcoxon tests were performed. FL: fetal livers, non-mos. HB: non-mosaic HB, HB: hepatoblastoma, pHCC: pediatric HCC, pFLC: pediatric fibrolamellar carcinoma, NT: non-tumor. Source data are provided as a Source Data file.

Supplementary Figure 19

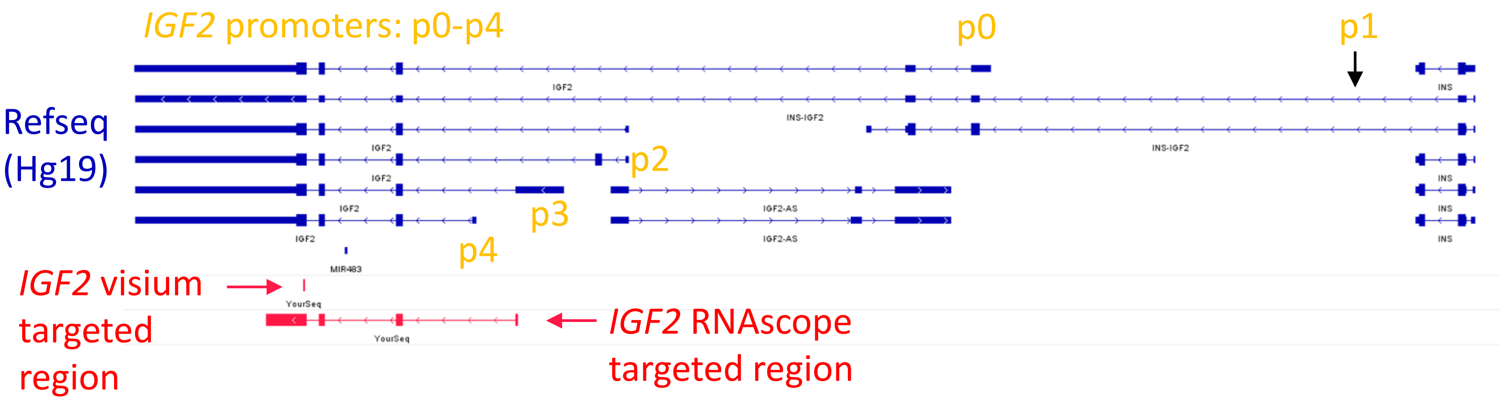

Supplementary figure 19. *IGF2* targeted regions in RNAscope and visium analyzes. The RNAscope and visium spatial transcriptomics targeted regions are indicated in Red and existing *IGF2* isoforms in RefSeq are shown in Blue. RNAscope of *IGF2* transcripts includes all *IGF2* isoforms including isoforms starting from promoters P0, P1, P2, P3 and P4. Approximately 20 probes (ZZ pairs) targeting *IGF2* transcript variant 1, mRNA (Biotechne, catalog #3594361) from nucleotide 692 to 2021. Visium probe is located in a region common to all *IGF2* isoforms indicating that visium *IGF2* quantifies all *IGF2* isoforms

Supplementary Table 1: Clinical, histological and molecular features of mosaic HB patients.

Mosaic patients features were compared with non mosaic patients (columns C-E).

HB with late 11p15 alteration were compared with non-late 11p15.5 HB : Mosaic, wt, not timed (F-H).

HB with 11p15.5 alteration (mosaic, late not timed) were compared with wild-type (I-K)

Source data are provided as a Source Data file

		**Mosaic HB vs non mosaic HB**			**HB 11p15.5 late vs non late 11p15.5 HB**			**HB 11p15.5 alt (mosaic, late, non timed) vs wt 11p15.5 HB**		
Association	pval age at surgery[1] (n=77)	mosaic patients (n = 13)	Non mosaic HB (n=64)	pval	HB 11p15.5 late (n=10)	non late 11p15.5 HB (n=64)	pval	HB 11p15.5 alt (n=55)	wt 11p15.5 HB (n=17)	pval
Age at surgery (Median years)	-	1.03	2.53	**$2.4.10^{-6}$§**	5.94	1.83	0.23§	1.52	2.57	0.13§
Sex (F)	**0.04**	6/13 (46%)	20/64 (31%)	0.30*	5/10 (50%)	21/54 (33%)	0.29*	20/45 (36%)	5/17 (29%)	0.59*
>30% Embryonal histology	0.54	5/13 (39%)	10/64 (16%)	0.13*	2/10 (20%)	11/64 (17%)	0.96*	12/55 (22%)	1/17 (6%)	0.14*
>30% Fetal histology	0.2	12/13 (92%)	55/64 (86%)	0.53*	8/10 (80%)	56/64 (88%)	0.48*	48/55 (87%)	15/17 (88%)	0.92*
>30% Mesenchymal histology	**$8.7.10^{-3}$**	4/13 (31%)	14/64 (22%)	0.49*	4/10 (40%)	14/64 (22%)	0.18*	15/55 (27%)	3/17 (18%)	0.42*
PRETEXT stade (1/2/3/4)	0.08	0/7/3/2	4/20/23/8	0.84±	1/7/0/1	3/20/26/9	**0.04±**	1/23/19/9	3/4/5/1	0.13
Tumor size >50mm	0.14	8/10 (80%)	29/42 (69%)	0.49*	7/8 (88%)	27/41 (66%)	0.22*	28/36 (78%)	5/12 (42%)	**0.01***
>1 nodule	**0.03**	1/9 (9%)	12/35 (34%)	0.17*	0/4 (0%)	13/37 (35%)	0.17*	10/29 (35%)	3/11 (27%)	0.46*
PRETEXT M	**$1.6.10^{-3}$**	1/12 (8%)	15/45 (27%)	0.16*	2/9 (22%)	14/58 (24%)	0.90*	13/52 (25%)	3/13 (23%)	0.89*
NFE2L2	0.12	0/11 (0%)	4/51 (9%)	0.34*	1/9 (1.1%)	3/53 (5.6%)	0.54*	4/51 (7.8%)	0/11 (0%)	0.34*
TERT	**$1.6.10^{-5}$**	0/11 (0%)	9/51 (0%)	0.13*	4/9 (44%)	5/53 (9.4%)	**0.006***	8/51 (16%)	1/11 (9%)	0.57*
Intra-tumor heterogeneity	0.22	11/12 (92%)	44/59 (75%)	0.44*	8/10 (80%)	47/61(77%)	0.84*	44/51 (86%)	11/18 (61%)	**0.02***
RNAseq group Liver progenitor	0.81	5/10 (50%)	20/51 (39%)	0.53*	5/9 (56%)	20/52 (39%)	0.34*	22/50 (44%)	3/11 (27%)	0.31*
RNAseq group Mesenchymal	**$5.3.10^{-3}$**	3/10 (30%)	6/51 (12%)	0.14*	1/9 (11%)	8/52 (15%)	0.74*	9/50 (18%)	0/11 (0%)	0.13*
RNAseq group Hepatocytic	**0.03**	2/10 (20%)	25/51 (49%)	0.09*	3/9 (33%)	24/52 (46%)	0.48*	19/50 (38%)	18/11 (73%)	**0.04***

F : female, PRETEXT M : Presence of metastasis at diagnosis

[1] Two-sided Mann-whitney Wilcoxon test or Kruskal-Wallis statistical test

* Two-sided Chi square

± Two-sided Trend Chi square

§ Two-sided Student's t-test

Red bold pvalues indicate significant associations

Supplementary Table 2: P1-P4 promoter coordinates (hg19) used in RRBS to determine the methylation status at 11p15.5 locus

	Chromosome	Start	End
P1	Chr11	2179456	2180571
P2	Chr11	2162039	2162932
P3	Chr11	2160014	2161212
P4	Chr11	2158570	2159332

Supplementary references

1. Bonder, M. J. *et al.* Genetic and epigenetic regulation of gene expression in fetal and adult human livers. *BMC Genomics* **15**, 860 (2014).
2. Cairo, S. *et al.* Hepatic stem-like phenotype and interplay of Wnt/beta-catenin and Myc signaling in aggressive childhood liver cancer. *Cancer Cell* **14**, 471–484 (2008).
3. Hooks, K. B. *et al.* New insights into diagnosis and therapeutic options for proliferative hepatoblastoma. *Hepatology* **68**, 89–102 (2018).
4. Nagae, G. *et al.* Genetic and epigenetic basis of hepatoblastoma diversity. *Nat Commun* **12**, 5423 (2021).
5. Hirsch, T. Z. *et al.* Integrated Genomic Analysis Identifies Driver Genes and Cisplatin-Resistant Progenitor Phenotype in Pediatric Liver Cancer. *Cancer Discov* **11**, 2524–2543 (2021).
