# Supplementary material for: Preneoplastic liver colonization by 11p15.5 altered mosaic cells in young children with hepatoblastoma
Source: Nat Commun. 2023 Nov 6;14:7122. doi: 10.1038/s41467-023-42418-9 (PMC10628292; doi:10.1038/s41467-023-42418-9)
Supplement: Supplementary file 2 — Description of Additional Supplementary Files [file 41467_2023_42418_MOESM2_ESM.pdf]

Description of Additional Supplementary Files

File Name: Supplementary Data 1
Description: **Cohort description**
Cohort of 131 individuals including 115 patients with pediatric liver tumor and 16 fetal livers. 165 corresponding tumor samples from 112 patients were analyzed.

File Name: Supplementary Data 2
Description: **Differential expression analysis between 7 fetal livers and 10 mosaic livers in Bulk RNAseq**
Limma package was used for differential expression analysis including a two-sided moderated t-test. Pvalues were adjusted with age at surgery using Benjamini Hochberg correction.

File Name: Supplementary Data 3
Description: **Differential expression analysis between 10 mosaic livers and 23 non mosaic livers in Bulk RNAseq**
Limma package was used for differential expression analysis including a two-sided moderated t-test. Pvalues were adjusted with age at surgery using Benjamini Hochberg correction.

File Name: Supplementary Data 4
Description: **Gene set enrichment analysis between 10 mosaic livers and 23 nonmosaic livers in Bulk RNAseq**
Limma package was used for differential expression analysis and p values were adjusted with age at surgery. A two-sided permutation test was perfomed using fgsea package to assess the significance of gene set enrichment. fgsea package was used for enrichment with 1000 permutations. Pvalues were adjusted for multiple testing using Benjamini-Hochberg correction.

File Name: Supplementary Data 5
Description: **Differentially expressed genes between mosaic and non-mosaic hepatocytes from all 3 mosaic 11p15.5 patients #4001, #3115 and #3559 in spatial transcriptomic analysis**
Differential expression analysis was performed using FindMarker Seurat function and a two-sided Wilcoxon rank-sum statistical test. Pvalues were adjusted for multiple testing using Bonferroni correction.

File Name: Supplementary Data 6
Description: **Gene set enrichment analysis between mosaic and non mosaic hepatocytes from all 3 mosaic 11p15.5 patients #4001, #3115 and #3559 in spatial transcriptomic analysis**
fgsea package was used for enrichment with 1000 permutations, minimal and maximal geneset size of respectively 25 and 200 genes. A two-sided permutation test was perfomed using fgsea package to assess the significance of gene set enrichment. Pvalues were adjusted for multiple testing using Benjamini-Hochberg correction.

File Name: Supplementary Data 7
Description: **Differential expression analysis of all cell types in snRNAseq.**
Differential expression analysis was performed with FindAllMarkers Seurat function and a Wilcoxon rank-sum two-sided statistical test. Pvalues were adjusted for multiple testing with Bonferroni correction. Genes were considered as differentially expressed with an adjusted pvalue ≤0.01 and when the gene was expressed in at least 25 percents of the cells in either population

File Name: Supplementary Data 8
Description: **Differentially expressed genes between mosaic and non-mosaic hepatocytes in patients #3115, #3559, #4001 and #2996 in snRNAseq**
Differential expression analysis was performed using FindMarker Seurat function and a Wilcoxon rank-sum two-sided statistical test. Pvalues were adjusted for multiple testing using Bonferroni correction. Genes were considered as differentially expressed with an adjusted pvalue ≤0.01 Only genes with a minimum log foldchange of 0.1 between the 2 conditions and an expression in at least 25 percents of the cells in either population were kept.

File Name: Supplementary Data 9
Description: **Functional enrichment between mosaic and non-mosaic hepatocytes in 4 patients analyzed in snRNAseq (#3115, #3559, #4001, #2996)**
Functional enrichment was performed using ToppGene suite and categories belonging to "Coexpression", "Computational", "Disease", "Pathway", "GO: Biological Process", "GO: Cellular Component", "GO: Molecular Function" and "Human Phenotype".
