# Supplementary material for: Preneoplastic liver colonization by 11p15.5 altered mosaic cells in young children with hepatoblastoma
Source: Nat Commun. 2023 Nov 6;14:7122. doi: 10.1038/s41467-023-42418-9 (PMC10628292; doi:10.1038/s41467-023-42418-9)
Supplement: Supplementary file 12 — Reporting Summary [file 41467_2023_42418_MOESM12_ESM.pdf]

nature portfolio

Corresponding author(s): Pr. Jessica Zucman-Rossi

Last updated by author(s): Oct 10, 2023

Reporting Summary

Nature Portfolio wishes to improve the reproducibility of the work that we publish. This form provides structure for consistency and transparency in reporting. For further information on Nature Portfolio policies, see our Editorial Policies and the Editorial Policy Checklist.

Statistics

For all statistical analyses, confirm that the following items are present in the figure legend, table legend, main text, or Methods section.

n/a	Confirmed	
☐	☒	The exact sample size (*n*) for each experimental group/condition, given as a discrete number and unit of measurement
☐	☒	A statement on whether measurements were taken from distinct samples or whether the same sample was measured repeatedly
☐	☒	The statistical test(s) used AND whether they are one- or two-sided<br>*Only common tests should be described solely by name; describe more complex techniques in the Methods section.*
☐	☒	A description of all covariates tested
☐	☒	A description of any assumptions or corrections, such as tests of normality and adjustment for multiple comparisons
☐	☒	A full description of the statistical parameters including central tendency (e.g. means) or other basic estimates (e.g. regression coefficient) AND variation (e.g. standard deviation) or associated estimates of uncertainty (e.g. confidence intervals)
☐	☒	For null hypothesis testing, the test statistic (e.g. *F*, *t*, *r*) with confidence intervals, effect sizes, degrees of freedom and *P* value noted<br>*Give P values as exact values whenever suitable.*
☒	☐	For Bayesian analysis, information on the choice of priors and Markov chain Monte Carlo settings
☒	☐	For hierarchical and complex designs, identification of the appropriate level for tests and full reporting of outcomes
☐	☒	Estimates of effect sizes (e.g. Cohen's *d*, Pearson's *r*), indicating how they were calculated

Our web collection on statistics for biologists contains articles on many of the points above.

Software and code

Policy information about availability of computer code

Data collection	We did not use any software for data collection
Data analysis	For Whole-genome sequencing (WGS) analysis we used:<br>-BWA tool for alignment<br>-Picard tools (http://broadinstitute.github.io/picard/) to remove PCR duplicates<br>-GATK tool for local indel realignment<br>-cgpBattenberg algorithm to reconstruct copy-number profiles (CNV)<br><br>For Whole-exome sequencing (WES) we used:<br>-BWA tool for alignment<br>-sambamba for the removal of deplicate reads<br>-Genome Alteration Print method to infer CNV profiles<br><br>MANTA software was used to detect large In-Frame deletions of CTNNB1 gene in WES and WGS.<br><br>For methylation profile analyses we used:<br>-BS_Seeker2 to perform alignment on hg38 genome version<br><br>For bulk RNAseq analyses we used :<br>-TopHat2 was used to align full Fastq files with human genome hg38<br>-DeSeq2 to normalize expression data through variance stabilization

-limma v3.62.2 for differential expression analyses between mosaic and non mosaic livers
-fgsea v1.20.0 for gene-set enrichment analyses
-heatmap.2 from gplots package v3.1.3 was used to perform supervised clustering analysis

For spatial transcriptomics and snRNAseq analyses we used:
-spaceranger v1.3.1 (mkfastq and count space ranger commands) for demultiplexing and read mapping
-seurat v4.0.1 for normalization, dimensionality reduction, clustering and differential expression analysis
-Harmony v0.1.1 for batch effect removal

For survival analysis we used:
-survminer v0.4.9
-survival v3.4.0
-rms v6.3.0

Data managment and visualization:
-readxl v1.4.1
-dplyr v1.0.9
-ggplot2 v3.3.6
-corrplot v0.92
-ggridges v0.5.3
-tidyr v1.2.1

R/RStudio v4.2.1 was used for analyses and plots

All custom scripts generated for this study are available on Github: https://github.com/FunGeST/Preneoplastic_liver_colonization.

For manuscripts utilizing custom algorithms or software that are central to the research but not yet described in published literature, software must be made available to editors and reviewers. We strongly encourage code deposition in a community repository (e.g. GitHub). See the Nature Portfolio guidelines for submitting code & software for further information.

Data

Policy information about availability of data

All manuscripts must include a data availability statement. This statement should provide the following information, where applicable:

- Accession codes, unique identifiers, or web links for publicly available datasets
- A description of any restrictions on data availability
- For clinical datasets or third party data, please ensure that the statement adheres to our policy

The raw data from this study, including WGS, WES, RNAseq, RRBS, snRNAseq and visium datasets have been archived in the EGA database under accession codes EGAS00001005108 (https://ega-archive.org/studies/EGAS00001005108), EGAS00001003837 (https://ega-archive.org/studies/EGAS00001003837) and EGAS00001006692 (https://ega-archive.org/studies/EGAS00001006692). Raw sequence data (FASTQ files) access is available upon successful application to the 'Data Access Committee' EGAC00001002924 according to the EGA general guidance. Access to these datasets is restricted to comply with the European data protection regulations and to protect the privacy and rights of individuals whose data may be included in these datasets. When the application is successful, access is granted for the duration of the approval project. Any utilization of the data for a different project will require prior approval through a new agreement. A response to all initial requests should be done in 6 weeks. The processed spatial transcriptomics and snRNAseq data can be found in the Figshare repository (https://doi.org/10.6084/m9.figshare.23552595), and source data are provided along with this paper. The fetal liver methylation publicly available data used in this study are available in the GEO database under accession code GSE61278 https://www.ncbi.nlm.nih.gov/geo/query/acc.cgi?acc=GSE6127821. The remaining data are available within the Article, Supplementary Information or Source Data file.

Human research participants

Policy information about studies involving human research participants and Sex and Gender in Research.

Reporting on sex and gender	We did not make specific observations applying to only one sex. Sex ratio was analyzed between mosaic and non mosaic 11p15.5 hepatoblastoma patients but this association was not significant.
Population characteristics	A cohort of 131 patients including 115 patients with pediatric liver cancers were collected from different French hospitals. In addition, 16 fetal liver samples from abortions were collected by the Centre de Ressources Biologiques (CRB) Bordeaux, in agreement with the institutional review board committee (approval number 2010-A00498-31). Several clinical and molecular features were analyzed such as age at diagnosis, location of the tumor, tumor histology, tumor stage (PRETEXT stage), tumor size and number of nodules. These features are reported in Supplementary Data 1.
Recruitment	These patients were mostly retrospectively retrieved except few recent cases.
Ethics oversight	The study was approved by the local Ethics Committee (CCPRB Paris Saint-Louis). Written informed consent was obtained in accordance with French legislation.

Note that full information on the approval of the study protocol must also be provided in the manuscript.

Field-specific reporting

Please select the one below that is the best fit for your research. If you are not sure, read the appropriate sections before making your selection.

☒ Life sciences ☐ Behavioural & social sciences ☐ Ecological, evolutionary & environmental sciences

For a reference copy of the document with all sections, see nature.com/documents/nr-reporting-summary-flat.pdf

Life sciences study design

All studies must disclose on these points even when the disclosure is negative.

Sample size	This study is a retrospective study where sample size was not predetermined. Given that hepatoblastoma is a rare disease, all good quality tissue from consent patient was included. Overall, this cohort is among the largest cohort of HB published.<br><br>*WES and WGS: Seventy-four non-tumor samples from hepatoblastoma (HB) patients were analysed in Whole-genome and Whole-exome sequencing (WES and WGS) to screen for 11p15.5 locus mosaicism (Supplementary Fig. 2). Moreover, 97 HB tumors were analyzed in WES/WGS.<br><br>*Bulk RNAseq: we compared the expression of 10 mosaic livers and 23 non mosaic livers that were sufficient to draw conclusions even after adjustment for age at surgery. In addition, gene expression of 123 HB tumor samples was included in this study.<br><br>*Targeted methylation profile from 73 non-tumor livers was assessed using MS-MLPA (Methylation-specific Multiplex Ligation-dependent Probe Amplification)<br><br>*Spatial transcriptomics: we performed visium (10x genomics) spatial transcriptomics in 3 patients with mosaic 11p15.5 alteration.<br><br>*Single-nucleus RNAseq: we performed snRNAseq (10x genomics) in the non-tumor liver from 3 patients with and 1 patient without 11p15.5 mosaisicm.
Data exclusions	*Three tumors with low tumor purity were removed from genomic analyses due to a high contamination by non-tumor cells that does not allow us to assess mutational and gene expression profile.<br><br>*For spatial transcriptomics, low quality spots were filtered out based on a very low number of genes detected in patient #3115 indicating a detachment of the tissue in a small area.<br><br>*For methylation analyses in MS-MLPA, we excluded one probe from analysis targeting IC1 (H19.11.001.976583) because its distribution did not discriminate between samples with and without gain of methylation IC1 (GOM IC1).<br><br>*In snRNAseq, cells with more than 5% mitochondrial genes were removed. We kept only cells with a minimum of 1,000 genes detected in each cell. We removed a cluster of cells with high mitochondrial genes, high ambient RNA and low features in each patient.
Replication	When possible, we sequenced multiple non-tumor and tumor samples for the same patient to assess mosaicism and tumor heterogeneity. Locus 11p15.5 mosaicism was detected using multiple complementary techniques including MS-MLPA, WGS or WES, in situ RNAscope hybridization. These complementary information allowed us to verify the reproducibility of our results.
Randomization	Not applicable
Blinding	Not applicable

Reporting for specific materials, systems and methods

We require information from authors about some types of materials, experimental systems and methods used in many studies. Here, indicate whether each material, system or method listed is relevant to your study. If you are not sure if a list item applies to your research, read the appropriate section before selecting a response.

Materials & experimental systems

n/a	Involved in the study
☐	☒ Antibodies
☒	☐ Eukaryotic cell lines
☒	☐ Palaeontology and archaeology
☒	☐ Animals and other organisms
☒	☐ Clinical data
☒	☐ Dual use research of concern

Methods

n/a	Involved in the study
☒	☐ ChIP-seq
☒	☐ Flow cytometry
☒	☐ MRI-based neuroimaging

Antibodies

Antibodies used

Immunostainings of β-catenin (BD Biosciences, Mouse IgG1, clone 14 ref #610154, 1/200) and glutamine synthetase (BD Biosciences, Mouse IgG2a, clone 6, ref #610517, 1/500) proteins were performed using DAKO Autostainer AS48L. Antigen retrieval was done at pH6 and pH9 for glutamine synthetase and β-catenin stainings respectively.

Validation

All the antibodies used in this study were validated for use in human specimens by the manufacturers.
-β-catenin (BD Biosciences, Mouse IgG1, clone 14 ref #610154)
"Application: Immunohistochemistry" (Manufacturer's website)

-glutamine synthetase (BD Biosciences, Mouse IgG2a, clone 6, ref #610517)
"Application:Immunohistochemistry (Tested During Development)" (Manufacturer's website)
